# The effects of cosolutes and crowding on the kinetics of protein condensate formation based on liquid–liquid phase separation: a pressure-jump relaxation study

**DOI:** 10.1038/s41598-020-74271-x

**Published:** 2020-10-14

**Authors:** Hasan Cinar, Roland Winter

**Affiliations:** Physical Chemistry I, Biophysical Chemistry, Faculty of Chemistry and Chemical Biology, TU Dortmund University, Otto-Hahn-Strasse 4a, 44227 Dortmund, Germany

**Keywords:** Biophysics, Chemistry

## Abstract

Biomolecular assembly processes based on liquid–liquid phase separation (LLPS) are ubiquitous in the biological cell. To fully understand the role of LLPS in biological self-assembly, it is necessary to characterize also their kinetics of formation and dissolution. Here, we introduce the pressure-jump relaxation technique in concert with UV/Vis and FTIR spectroscopy as well as light microscopy to characterize the evolution of LLPS formation and dissolution in a time-dependent manner. As a model system undergoing LLPS we used the globular eye-lens protein γD-crystallin. As cosolutes and macromolecular crowding are known to affect the stability and dynamics of biomolecular condensates in cellulo, we extended our kinetic study by addressing also the impact of urea, the deep-sea osmolyte trimethylamine-*N*-oxide (TMAO) and a crowding agent on the transformation kinetics of the LLPS system. As a prerequisite for the kinetic studies, the phase diagram of γD-crystallin at the different solution conditions also had to be determined. The formation of the droplet phase was found to be a very rapid process and can be switched on and off on the 1–4 s timescale. Theoretical treatment using the Johnson–Mehl–Avrami–Kolmogorov model indicates that the LLPS proceeds via a diffusion-limited nucleation and growth mechanism at subcritical protein concentrations, a scenario which is also expected to prevail within biologically relevant crowded systems. Compared to the marked effect the cosolutes take on the stability of the LLPS region, their effect at biologically relevant concentrations on the phase transformation kinetics is very small, which might be a particular advantage in the cellular context, as a fast switching capability of the transition should not be compromised by the presence of cellular cosolutes.

## Introduction

The maintenance of cellular function depends on a high degree of organization that prevails through different structural levels, from biomolecular complexes to larger subcellular structures such as organelles. In recent years it has become clear that assembly processes based on liquid–liquid phase separation (LLPS) of protein and protein-nucleic acid mixtures, acting as membraneless organelles, play an important role in cellular self-assembly processes^1–8^. Examples of such membrane-less organelles are cytoplasmic granules, nucleoli, clusters of proteins involved in signaling, and postsynaptic densities, which are protein-enriched cellular compartments beneath postsynaptic membranes^1–6^. One advantage of such membraneless compartments is that their biological function can be switched on and off relatively quickly compared to the build-up of lipid-based membrane envelopes.

The existence and location of all phase transitions, including complex biomolecular mixtures undergoing LLPS, depend on the fundamental thermodynamic variables, i.e. temperature, pressure and the concentrations (activities) of the constituents. The thermodynamic basis of such more-component system describes the equilibrium behavior of the system only, but does not provide any information about the kinetics of the underlying phase separation process. Such information is required to fully comprehend the role of LLPS-driven biological compartmentalization processes, however. The kinetics of a particular LLPS formation and dissolution is expected to meet the pertinent timescale required for the biochemical processes taking place in such assemblies. For signaling processes and enzymatic reactions requiring the co-localization of the reactants in the liquid condensates, the formation or dissolution of the LLPS is generally expected to be the rate-limiting step. As an example, stress granules need to form rapidly when an organism is exposed to sudden external stress conditions. Therefore, the biological implications of thermodynamically driven liquid–liquid phase transitions, such as those encountered in liquid protein or protein/nucleic acid condensates, cannot be appreciated without accounting for their kinetics. This kinetic aspect is the focus of this study.

**Figure 1:**
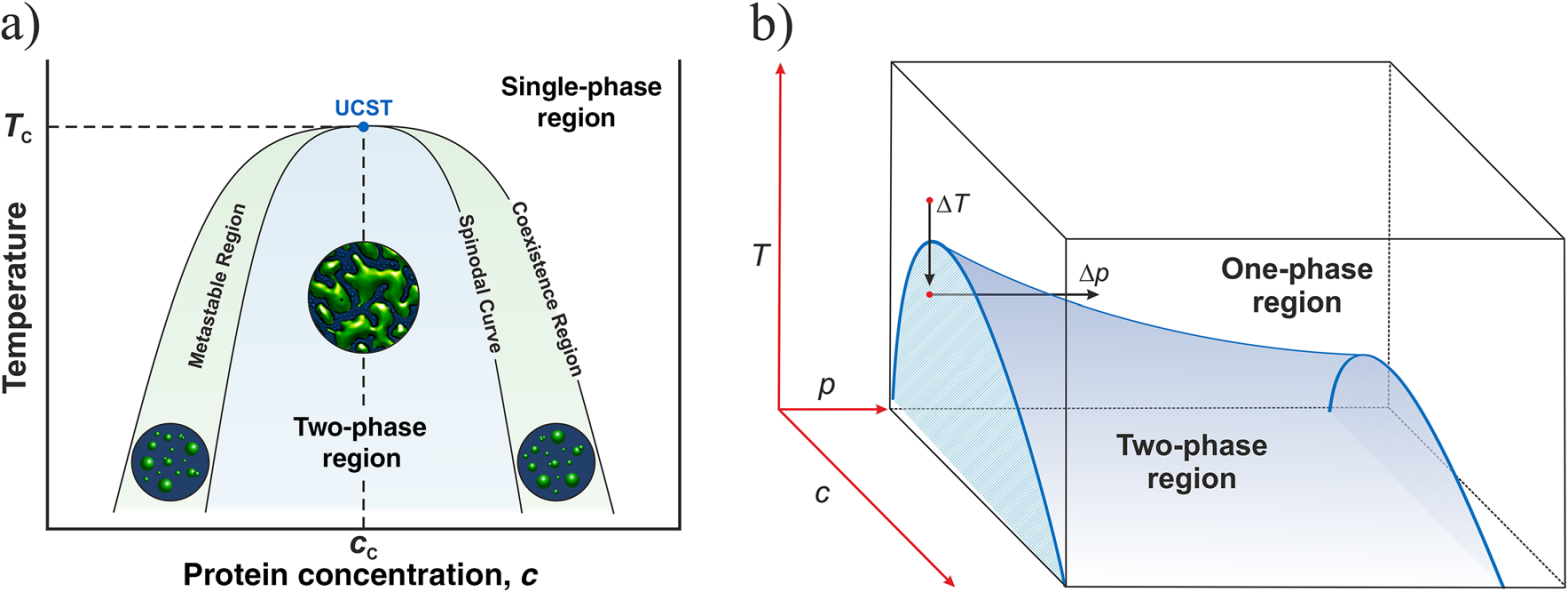
(**a**) Representative temperature-concentration phase diagram for a partially miscible binary liquid mixture (e.g., a highly concentrated lysozyme or γ-crystallin solution) with an upper critical solution point (UCST: upper critical solution temperature at critical protein concentration, cc), the diagram was reproduced from Reference^11^. The phase separation kinetics may vary widely in time, depending on the region of the phase diagram crossed, and across different systems. Phase separation by droplet nucleation and growth occurs in the metastable regions, phase separation by spinodal decomposition in the critical point region and below the spinodal curve, where highly dynamic phase-separated domains on all length scales emerge with essentially no nucleation barrier. At very high protein concentrations, an interplay between spinodal decomposition and dynamical arrest (with gel-like properties) may be observed, such as in lysozyme solutions at high ionic strength and low temperatures^7,8^. (**b**) Schematic of the *T*,*p*,*c* phase diagram of a LLPS system such as of *γ*-crystallin with an UCST (*c*, protein concentration). The arrows indicate the direction of temperature- or pressure-jumps for studying the kinetics of the phase transformation.

Prerequisite for the kinetic studies of the LLPS system is the knowledge of the phase diagram of the system at the various solution conditions. Inasmuch as temperature dependence is concerned, most protein condensates exhibit an upper critical solution temperature (UCST) in that LLPS occurs below a critical temperature *T*_c_ (i.e., *T*_c_ = UCST, the protein concentration at *T*_c_ is its critical concentration, *c*_c_; see Fig. 1). However, systems with lower critical solution temperature (LCST) or even reentrant phase separation systems are known as well^1,9,10^.

Recently, we were able to show that some protein systems undergoing LLPS are also very sensitive to pressure. In some cases, such as for γ-crystallin, pressures of several hundred bar were shown to be able to induce the transformation from the phase-separated to the homogeneous solution state^9,10,12^. Here, we applied, to our knowledge for the first time, the pressure-perturbation approach to study also the kinetics of the LLPS process of a protein from the family of crystallins, which belong to the family of mammalian lens proteins and are particularly concentrated in the cytoplasm of the eye lens cells^13,14^. Perturbation by high pressure has become increasingly popular in the field of protein folding due to the specific effects of pressure on non-covalent bonds, mainly hydrophobic and electrostatic interactions, and on protein systems displaying a significant amount of packing defects or void volume^15–18^. In addition, the necessary technical development for spectroscopic and kinetic investigations under high pressure has much evolved and is available now in several laboratories^19–22^. The pressure-jump approach has several advantages over other techniques for studying the kinetics of phase transformations, such as temperature- or pH-jumps. Using pH-jumps, the protein's surface charge distribution and hence stability is altered. Using temperature-jumps, intermolecular forces and the conformational dynamics of the protein are prone to change. Owing to the aggregation propensity of proteins at higher temperatures, temperature-jump experiments are often not reversible. Pressure is a very mild perturbating agent^15–18^. Pressure-jumps can be conducted in both phase transition directions (upward and downward), in the absence of thermal effects, and they are generally—as shown here—fully reversible. Pressure experiments are controlled by the volumetric (packing) properties of the system, which depend also strongly on the solvation conditions of the biomolecules, and do not require a change in thermal energy of the system. According to Le Châtelier's principle, the driving force is an overall reduction of volume, e.g. by filling void volume with solvent or due to electrostriction, i.e. hydration of charges upon dissociation of ion pairs^15–18,20–22^. Further, pressure propagates rapidly (with the speed of sound) so that sample inhomogeneity is no problem^15,18^.

We chose the protein γD-crystallin, which is a major component of the mammalian lens proteins and a good model system for globular proteins undergoing LLPS^23^. The concentrations of lens proteins reach values as high as several hundred mg mL^−1^, and their dense and homogeneous packing defines the refractive index of the eye lens, which needs to be transparent^13,14^. Density and concentration fluctuations imposed by LLPS and/or protein crystallization induce light scattering and hence loss of lens opacity, which is the cause of cold cataract. In fact, highly concentrated solutions of crystallins are known to undergo phase separation, however at low temperatures only, but their UCST depends on the protein’s specific amino acid sequence and can change significantly through mutations^13,14,23,24^. In view of the charged and hydrophobic residues on the folded γD-crystallin surface, the phase-separated condensed phase at low temperature is stabilized by transient electrostatic, hydrophobic, and van der Waals interactions among neighboring protein molecules^12^.

In this study, we have utilized UV/Vis turbidity measurements, FTIR spectroscopy and light microscopy in various high-pressure sample cells to study the phase properties and transformation kinetics of the γD-crystallin LLPS system. As cosolvents and crowding agents are common components of the cellular milieu and are known to affect the relative stabilities of biomolecular systems including protein condensates^10,12,25–27^, we extended our kinetic study by addressing also the impact of urea, trimethylamine-*N*-oxide (TMAO) and the crowding agent Ficoll on the phase stability and transformation kinetics of the system. Interestingly, TMAO is upregulated in organisms thriving in the deep sea at high pressures of several hundred bar. For that reason, TMAO is believed to serve as a pressure counteractant, or a "piezolyte", and its physico-chemical properties have been extensively studied, recently^28–31^.

## Results and discussion

### Protein stability

A first prerequisite for the *p*-jump experiments to determine the kinetics of LLPS is that the protein is stable in the whole temperature and pressure range under consideration. Hence, to ascertain whether the native protein fold of γD-crystallin is retained up to the highest pressure used in our LLPS study, Fourier-transform infrared (FTIR) spectroscopy has been used. FTIR spectroscopy probes changes in secondary structure elements of the protein. The pressure-dependent structural properties of *γ*D-crystallin were studied in the homogeneous phase at 25 °C and in the phase-separated region at 4 °C. The rationale of this investigation was also to detect possible structural changes that could take place during the transition from the homogeneous phase to the phase-separated state. Furthermore, the data also allow us to determine if the LLPS has a stabilizing or destabilizing effect on the structural stability of the protein.

**Figure 2:**
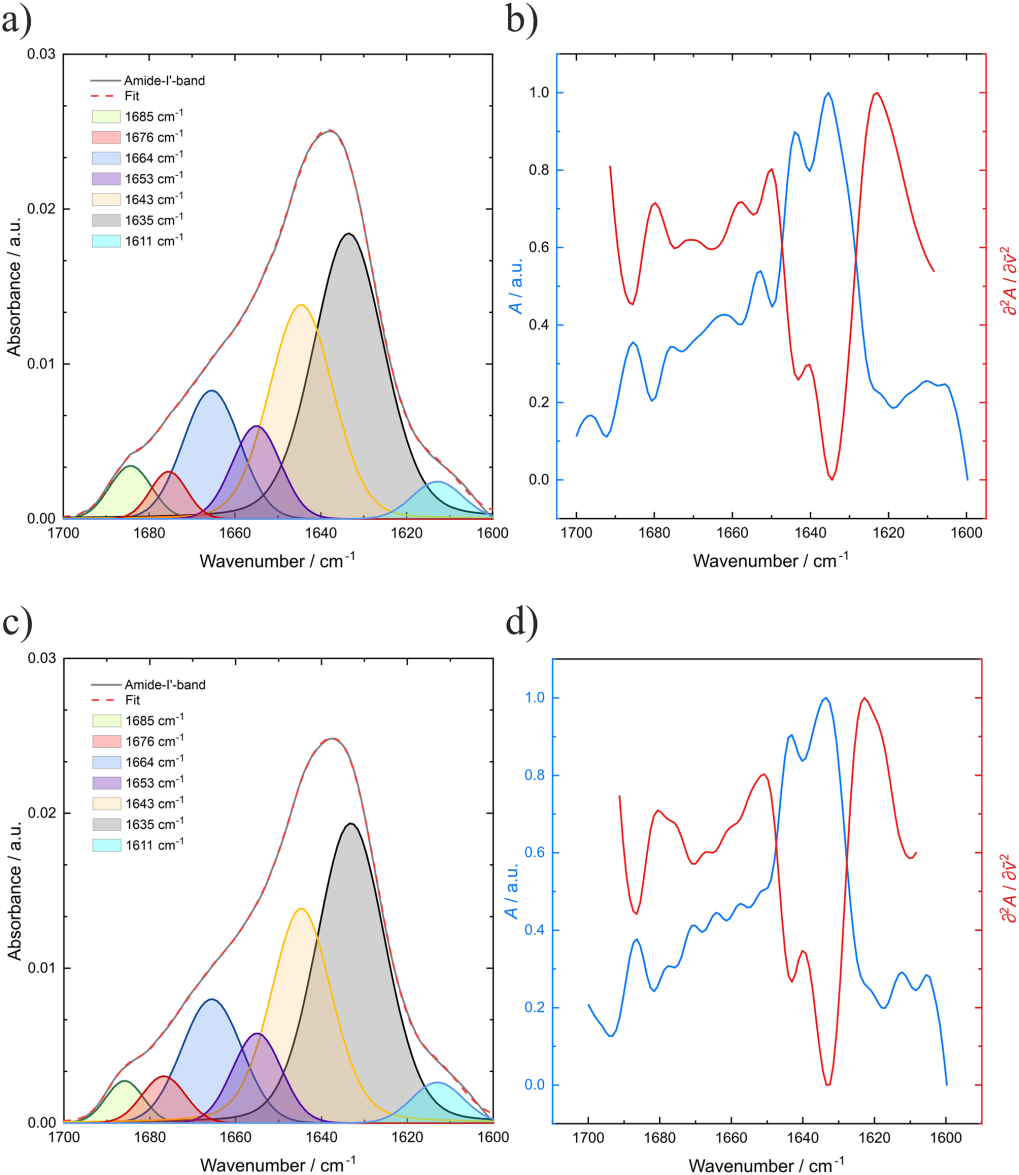
Deconvoluted FT-IR amide I′ band of *γ*D-crystallin into its subcomponents at (**a**) 24 °C and (**c**) 4 °C at ambient pressure. (**b**) FSD treated spectra and the second derivative of *γ*D-crystallin at (**b**) 24 °C and (**d**) 4 °C at ambient pressure.

Figure 2 shows the deconvoluted FTIR absorption spectra of *γ*D-crystallin as a function of pressure at 25 °C (in Figure SI 1, we show the original FT-IR spectra and difference spectra in the pressure range 1–10,000 bar). The amide I′ band recorded here has a maximum at 1639 cm^−1^ at 1 bar. With increasing pressure, a shoulder forms at ~ 1620 cm^−1^, indicating a minor pressure-dependent structural change at high pressures, only. When comparing the pressure-dependent FTIR absorption spectra of γD-crystallin at 4 °C with those at 25 °C (Fig. 2c,d), no significant differences can be seen. Second derivative and Fourier self-deconvolution (FSD) of the amide I′ band allowed us to determine relative changes of secondary structure elements. The minima of the second derivative and maxima of the FSD treated spectra indicate the band positions of the corresponding secondary structure elements (Fig. 2). The subbands at 1685 cm^−1^, 1676 cm^−1^ and the major band 1635 cm^−1^ can be assigned to β-sheet structures. The band at 1664 cm^−1^ is associated with β-turns and the band at 1653 cm^−1^ is assigned to α-helical structures. The band at 1643 cm^−1^ can be associated with disordered structures, while the small band at 1611 cm^−1^ can be represented by exposed β-sheets. The direct comparison of the deconvoluted FT-IR amide I′ band at 4 °C and 24 °C demonstrates that there are no significant differences in the secondary structural fractions (Table SI 1). This indicates that the LLPS formation is not associated with changes in the secondary structure of the protein.

**Figure 3:**
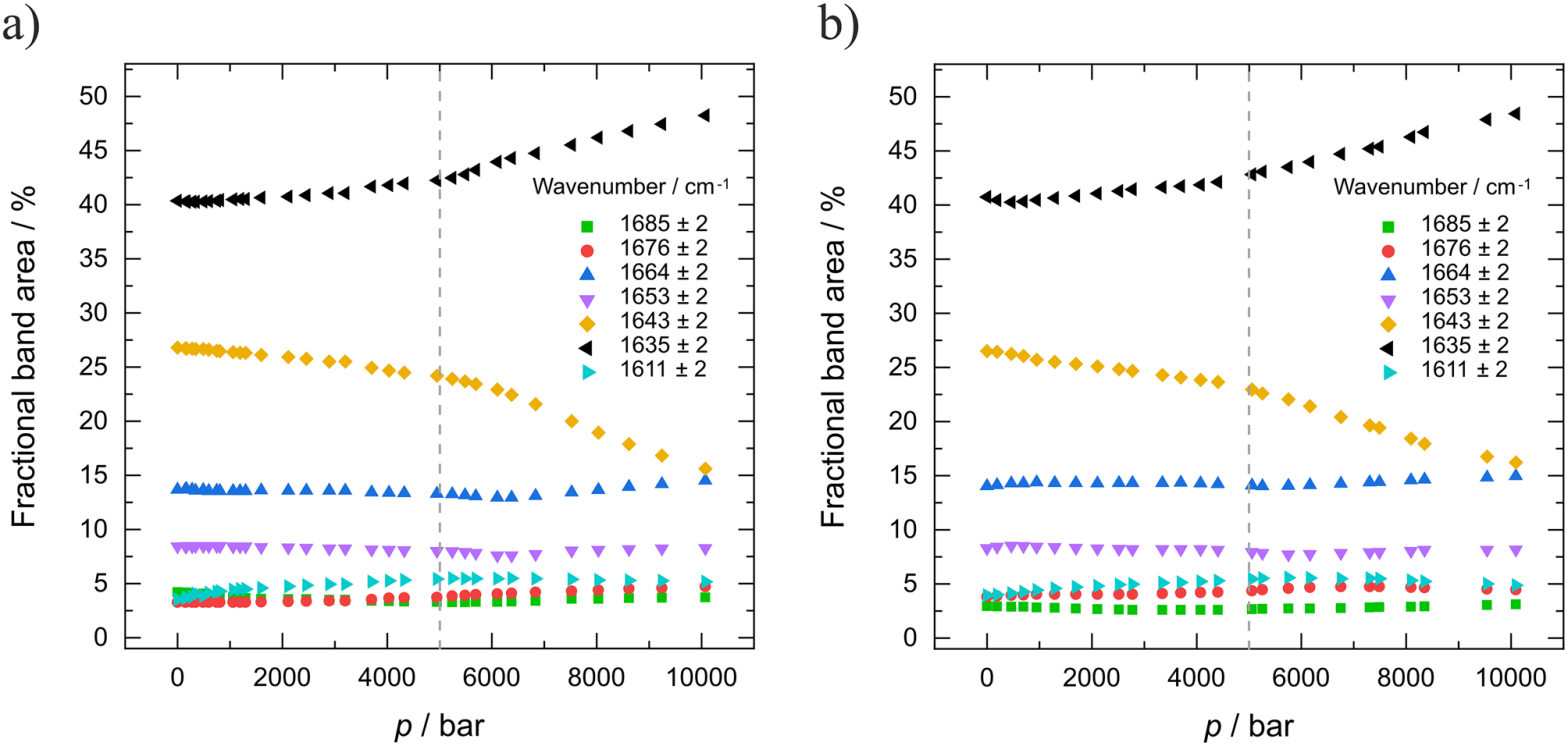
Secondary structure analysis of *γ*D-crystallin (PDB: 1H4A). The fractional band areas of secondary structure elements at (**a**) 24 °C and (**b**) 4 °C are shown as a function of pressure.

The detailed secondary structural analysis (Fig. 3) shows that the phase-separated system has neither a stabilizing nor a destabilizing effect on the protein during the pressure increase. Between about 1 and 5 kbar, *γ*D-crystallin undergoes a very small conformational drift only, where the amount of β-sheet structures seems to increase slightly at the expense of disordered structures. This trend is more pronounced at pressures beyond 5 kbar, where these changes amount to about 10% at 10 kbar. A pressure-induced unfolding of the protein would have resulted in the appearance of a pronounced subband at ~ 1643 cm^−1^^15,20^ representing random coil conformations (for illustration, Figure SI 1e shows the temperature-induced unfolding of the protein). Overall, *γ*D-crystallin is very pressure stable and no unfolding takes place even at 10 kbar. In agreement with earlier fluorescence studies, no significant secondary structural changes were observed up to about 5 kbar at 4 °C and 25 °C^12^. Monomeric proteins typically unfold at pressure between 4 and 8 kbar^15–18,32^. Hence, in the whole pressure range covered in the pressure-jump kinetic studies presented here (below 1 kbar), no structural changes of the protein take place.

### Phase coexistence curve and cosolute effects

As a second prerequisite for the kinetics experiments, the phase diagram of the LLPS system had to be established for the different cosolute conditions. The LLPS was examined by monitoring the turbidity (apparent absorption, *A*) through light scattering at 400 nm using a UV/Vis spectrometer equipped with a high-pressure cell. The temperature of the sample cell was controlled by an external water thermostat (for details see the “Experimental procedures” section). The protein concentration of *γ*D-crystallin was varied between 20–150 mg mL^−1^. For the preparation of the samples, Tris buffer with pH = 7.4 was chosen, which was also used for the pressure dependent measurements. To determine the temperature-induced cloud points, the respective samples of different concentrations were cooled down stepwise (in 0.5 °C-steps) until an increase in absorbance was observed.

**Figure 4:**
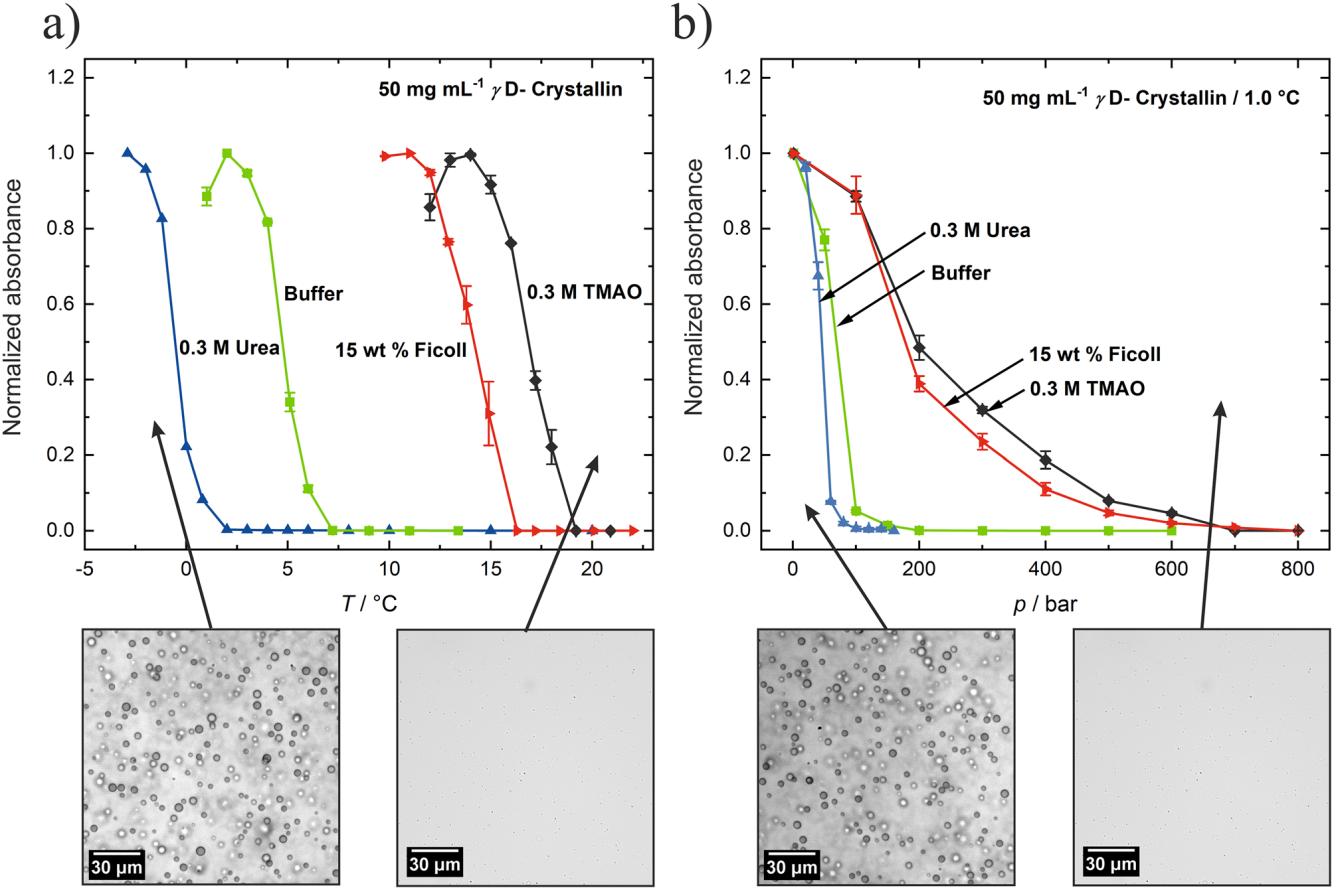
Representative UV/Vis absorption (turbidity) data at 400 nm of a 50 mg mL^−1^ solution of *γ*D-crystallin as a function of (**a**) temperature in buffer (50 mM TRIS, 150 mM NaCl, pH 7.4), 0.3 M urea, 15 wt% Ficoll and 0.3 M TMAO, and (**b**) pressure at *T* = 1 °C. Bottom: light microscopy snapshots of *γ*D-crystallin representing the phase-separated and the homogeneous state of the solution. The absorption data are normalized to their maximum values (1.0); the unnormalized data are shown in Figure SI 2).

As representative examples, Fig. 4a shows the UV/Vis absorption data of γD-crystallin at 50 mg mL^−1^ as a function of temperature under atmospheric pressure for three representative solution conditions: neat buffer, 0.3 M TMAO and 15 wt% of the macromolecular crowding agent Ficoll. The γD-crystallin at ambient pressure exhibits a temperature-induced cloud point, *T*_cloud_, at ~ 6 °C. Above ~ 7 °C, a homogeneous phase ensued. Addition of 0.3 M urea leads to a decrease of *T*_cloud_ to 1 °C. Conversely, addition of 15 wt% Ficoll and 0.3 M TMAO lead to an increase of *T*_cloud_ to ~ 15 °C and ~ 18 °C, respectively. The light microscopy pictures shown illustrate the phase-separated and homogeneous state of the buffer solution. The appearance of the droplet phase, i.e. the size and shape of the droplets, does not seem to change significantly with the different cosolutes. Figure 4b depicts pressure-dependent absorbance data at *T* = 1 °C for these four different solution conditions. As can be clearly seen, the addition of cosolutes causes a shift in the pressure needed to move from the demixed to the homogeneous state. Urea shifts the droplet-to-homogeneous-phase transition to lower pressure. Conversely, in the Ficoll and TMAO solutions significantly higher pressures are needed to induce the transition, i.e., the compatible cosolvent TMAO and the crowding agent Ficoll stabilize the droplet phase of γD-crystallin, whereas the chaotropic agent urea has the opposite effect.

**Figure 5:**
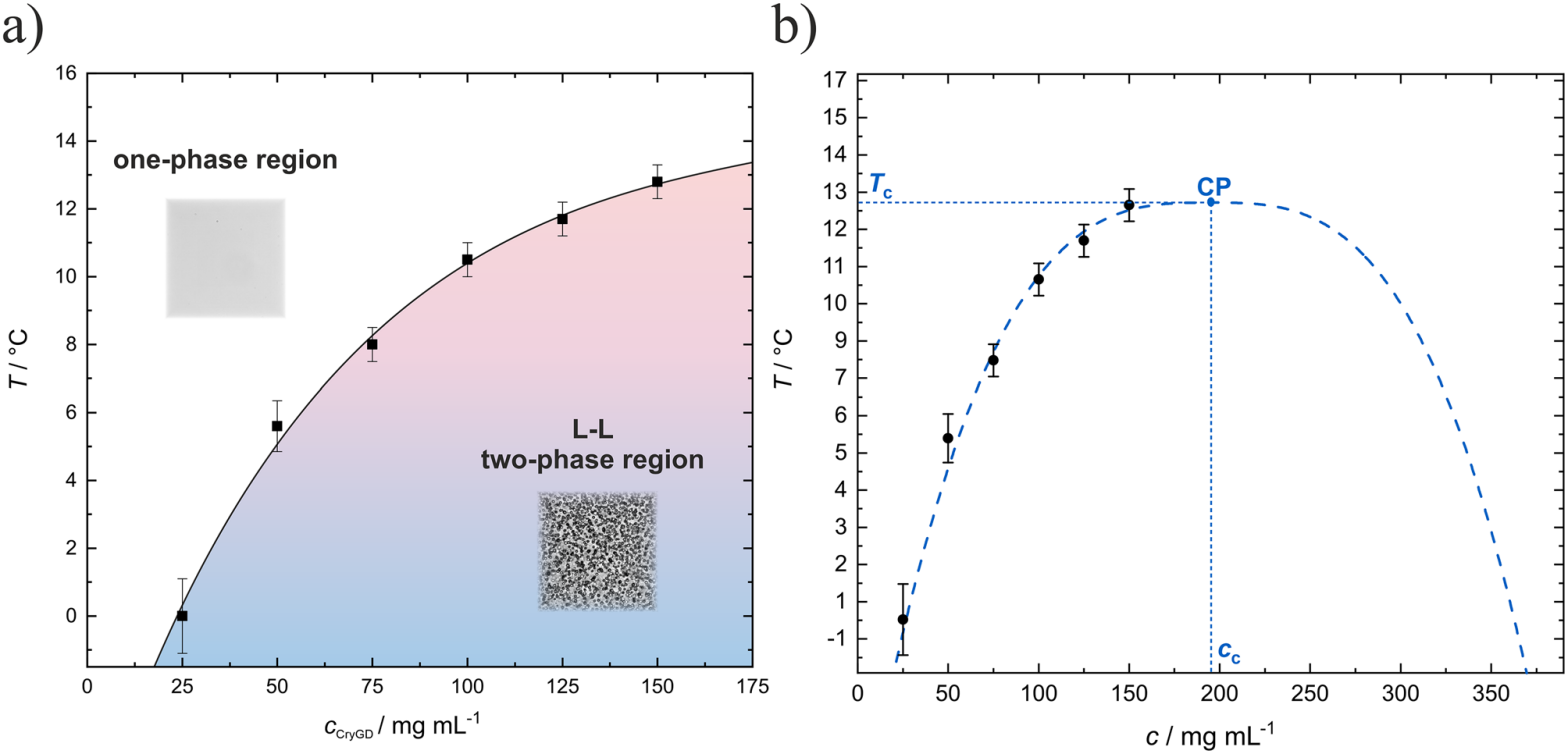
(**a**) *T, c*_CryGD_-phase diagram of *γ*D-crystallin in neat buffer at ambient pressure. The LLPS region is indicated by colored shading (red to blue shading: decreasing temperature). The homogeneous and phase separated regions are highlighted by light microscopy images. The data points for cloud point determination are from the turbidity experiments. (**b**) Fit to the coexistence curve using Eq. (1) with the critical parameter *β* = 0.33 describing the shape of the coexistence curve in the critical point region.

The protein concentration dependent measurements show that the cloud point temperature is strongly dependent on the protein concentration. For a protein concentration of 25 mg mL^−1^ in neat buffer solution, a cloud point temperature of 0.0 ± 1.1 °C was obtained, and a concentration of 150 mg mL^−1^ shifts *T*_cloud_ to 12.8 ± 0.5 °C. While a significant temperature increase can be observed at lower concentrations, the temperature difference decreases with increasing concentration. Figures 5a shows the *T*-*c*_CryGD_ phase coexistence curve of the LLPS region as deduced from these measurements for neat buffer.

Measurements of the *T*-*c*_CryGD_ two-phase coexistence region at higher protein concentrations become more difficult owing to the drastically increased viscosity of the solution, finally leading to dynamic arrest and gel formation. Following a study by Thomson et al.^33^, the shape of the two-phase coexistence curve may be fitted in a way that a theoretical treatment would predict of such a phase separation in the vicinity of the critical point. We have, therefore, performed a fit of the coexistence curve to the expression

1 $$\left| {\frac{{c - c_{{\mathrm{c}}} }}{{c_{{\mathrm{c}}} }}} \right| = w\left| {\frac{{T_{{\mathrm{c}}} - T}}{{T_{{\mathrm{c}}} }}} \right|^{\beta }$$

where *c* = *c*_CryCD_ is the concentration of either phase in mg mL^−1^, *c*_c_ is the critical concentration, *T*_c_ is the critical temperature in K, and *w* is a dimensionless quantity that characterizes the width of the coexistence curve. With the critical parameter *β *≈ 0.33 for the 3 D Ising model^34,35^, we obtain values of *c*_c_ = 195 ± 14 mg mL^−1^, *T*_c_ = 13.0 ± 0.5 °C, and *w* = 2.4 ± 0.1. The critical point (CP) and coexistence curve corresponding to these parameters and to Eq. (2) are shown in Fig. 5b.

**Figure 6:**
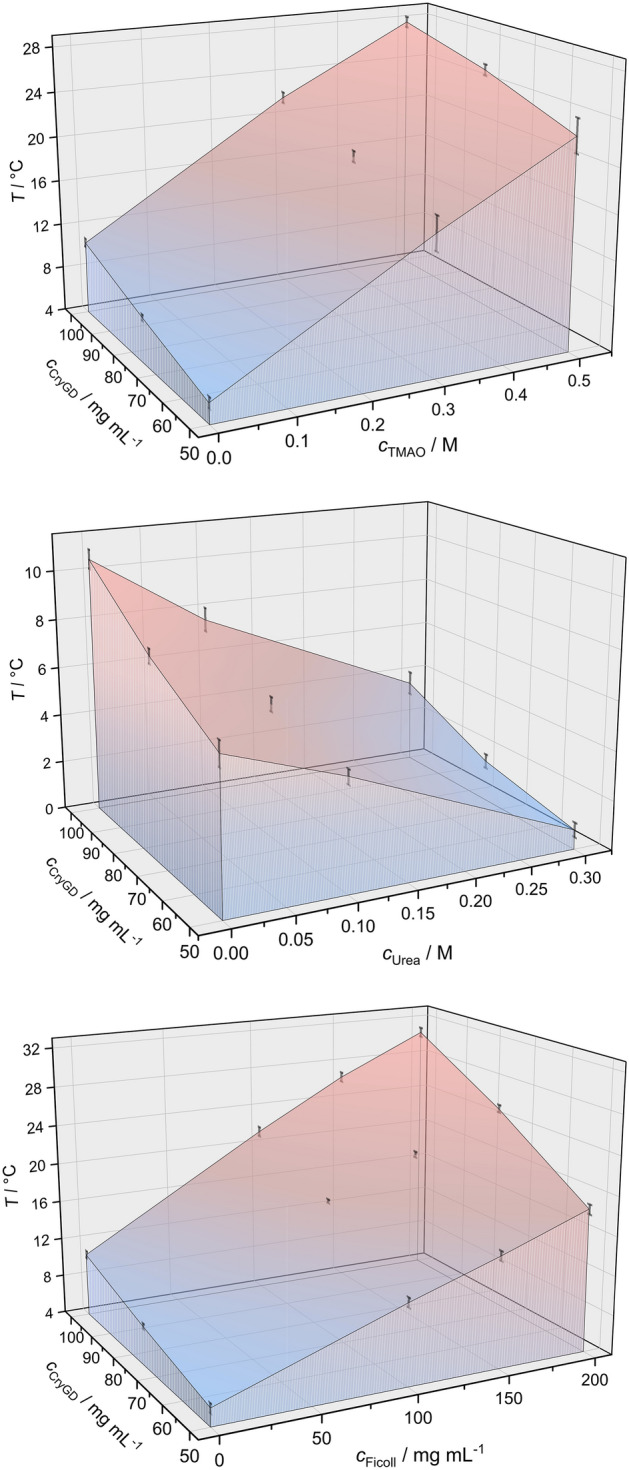
Effect of temperature, cosolute (TMAO, urea, Ficoll) and protein concentration on the LLPS of *γ*D-crystallin. The data points for cloud point determination were taken from the turbidity experiments (error bars are obtained from three independent measurements).

Figure 6 depicts 3D plots of the phase diagram for all cosolute solutions measured, TMAO, urea and Ficoll, as a function of temperature and γD-crystallin concentration. Addition of the ecompatible osmolyte TMAO increases the cloud point temperature of *γ*D-crystallin significantly, in agreement with an earlier study of our lab^10,12^. For example, by adding 0.5 M TMAO, *T*_cloud_ further shifts to 27.7 °C in the presence of 100 mg of the protein, i.e. by almost threefold. Conversely, addition of the chaotrope urea suppresses the cloud point temperature. *T*_cloud_ decreases to ~ 3.5 °C in the presence of 0.3 M urea for a protein concentration of *c* = 100 mg mL^−1^. Remarkably, in 0.5 M urea solution, LLPS in *γ*D-crystallin was not observed anymore above the freezing point of the solution. An even more drastic increase in cloud point temperature compared to TMAO is observed upon addition of the crowding agent Ficoll (Fig. 6c). By adding 200 mg mL^−1^ Ficoll, which corresponds to a typical macromolecular crowding situation encountered in biological cells, *T*_cloud_ increases from 10.5 to 30.5 °C in the presence of 100 mg of the protein.

### Phase transition kinetics

Based on the knowledge of the phase boundaries of the two-phase system of the protein at the different solution conditions, the kinetic measurements could now be carried out. Fast pressure (*p*)-jumps with high temporal resolution (deadtime 50–100 ms) across the phase boundaries as applied here are perfectly suited to study the kinetics of the liquid–liquid phase separation, i.e. the overall protein droplet formation or dissolution. Temperature-jumps using laser technology could principally also be used. However, depending on the sample's characteristics and relaxation times involved, often the temperature profile of the process is detected and not the system's relevant process. Also, a temperature change to a lower temperature is tricky to carry out. As pressure dependent processes below the 10 kbar range on proteins are generally fully reversible, bidirectional *p*-jumps as used here are superior in our case (for experimental details, see below).

**Figure 7:**
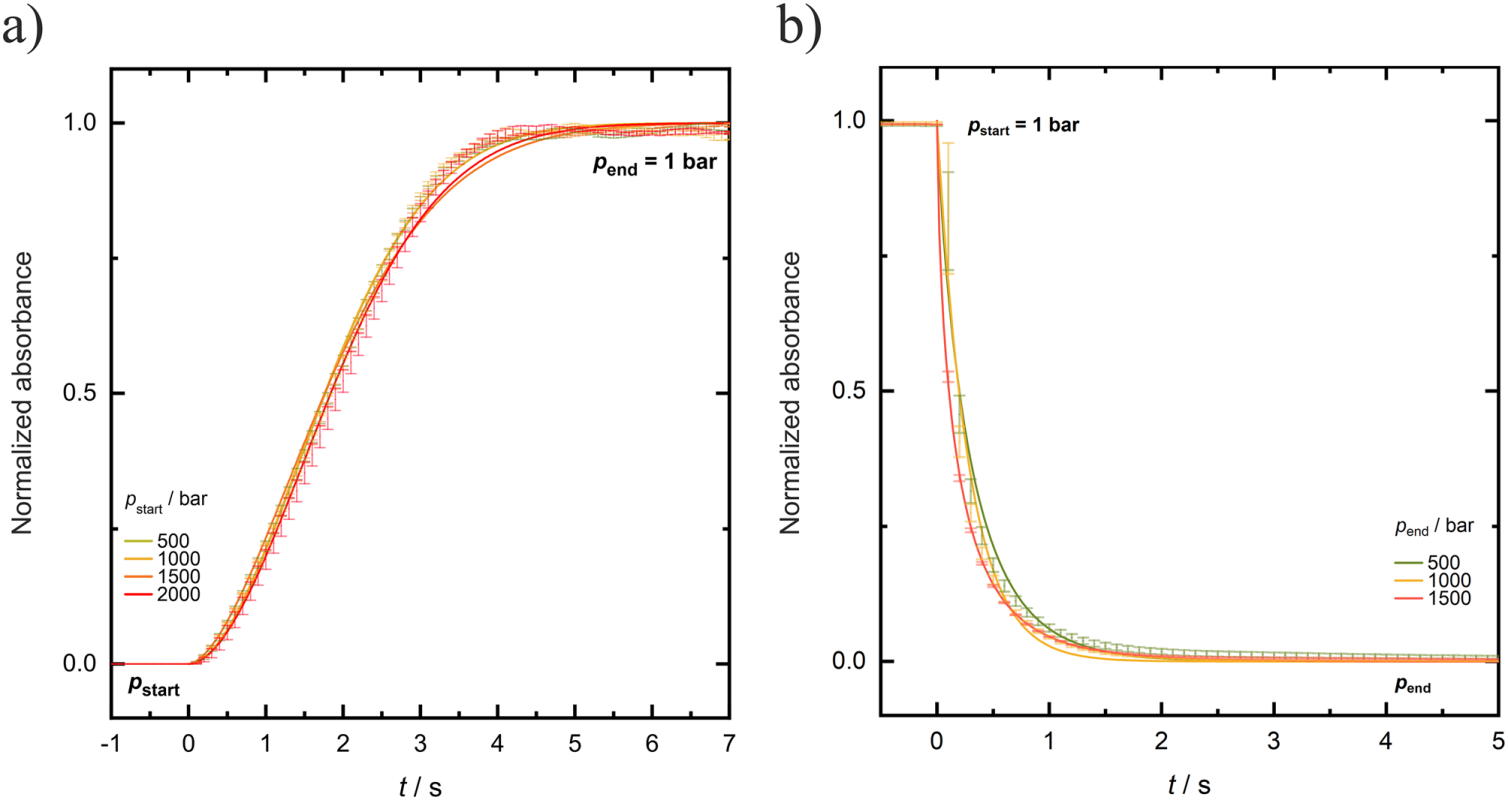
Time course of the absorption (turbidity), *A*, of a 50 mg mL^−1^ *γ*D-crystallin solution in neat buffer at *T* = 3 °C after *p*-jumps of variable amplitude; (**a**) from high pressure to ambient pressure (depressurization, LLPS formation) and (**b**) from 1 bar to high pressure (pressurization, vanishing of the two-phase region). The absorption data are normalized to their maximum values (1.0), wherein an absorbance of ~ 1 represents the phase-separated equilibrium state of *γ*D-crystallin in the LLPS state at atmospheric pressure.

Figure 7a depicts the absorbance data of γD-crystallin at a concentration of 50 mg mL^−1^ at 3 °C following a rapid *p*-jump at time *t* = 0 from different pressures above 500 bar to ambient pressure (1 bar), i.e. in the depressurizing, LLPS-forming direction. The absorbance (turbidity), *A*, is seen to increase rapidly when entering the LLPS region from the homogeneous phase, and levels off after about 4.4 s when reaching an equilibrium state. Figure 7b shows the *p*-jump data in the opposite, i.e. pressurization direction, which reveal an about three times faster kinetics. Measurements have also been taken at different temperatures. All data are summarized in Tables SI 2.1 and 2.2. Figure SI 3 shows a series of successive pressure cycles across the two-phase region of *γ*D-crystallin, demonstrating the switching capability and full reversibility of the transition.

To quantify the kinetic data, the kinetic profiles were fitted to the Johnson–Mehl–Avrami–Kolmogorov (JMAK) function. Following Eq. (2), $\theta_{{\mathrm{d}}} \propto A = 1 - \exp ( - kt)^{n}$ when entering the LLPS region, and $\theta_{{\mathrm{d}}} \propto A = \exp ( - kt)^{n}$ when entering the homogeneous phase; *n* is the Avrami exponent, and *k* an apparent rate constant (owing to the normalization of the absorption data, the prefactor in Eq. (2) is *C* = 1)*.* From the time-lapse kinetic data, the overall transition time, *t*_tr_, i.e. the time for completion of the phase transition and reaching an equilibrium state, and the half-life time, *t*_1/2_, the time where half of the absorption intensity changes are reached, were determined. Experiments have also been carried out at different temperatures. All data are reported in the Table SI 2.

At *T* = 3 °C, the time constants in the depressurization direction, from the homogeneous phase to the LLPS region, amount to *t*_tr_ = 4.4 ± 0.3 s and *t*_1/2_ = 1.8 ± 0.1 s. Interestingly, changing the *p*-jump amplitude has no significant effect on the kinetics of phase droplet formation. Also a higher protein concentration (75 mg mL^−1^) yielded similar results. A 5 °C increase of temperature results in a slight decrease (about 1.0 s in *t*_tr_ and 0.3 s in *t*_1/2_,) of the time constants. The comparison of Figs. 7a,b reveals that in the pressurization direction, i.e. from the LLPS region to the homogeneous phase, the kinetics is faster, with time constants of *t*_tr_ = 1.5 ± 0.2 s and *t*_1/2_ = 0.2 ± 0.1 s at *T* = 3 °C.

**Figure 8:**
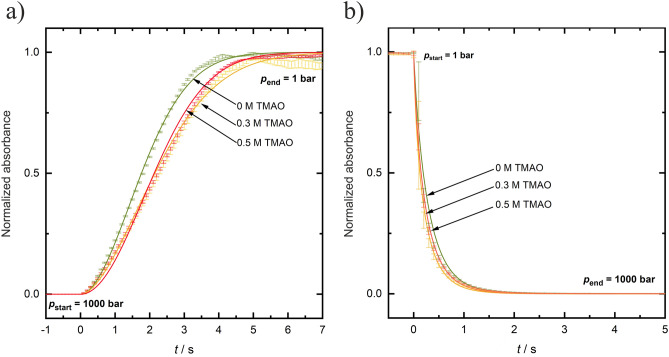
Effect of TMAO concentration on the kinetics of LLPS formation of a 50 mg mL^−1^ *γ*D-crystallin solution at *T* = 3 °C (**a**) upon decompression (LLPS formation) and (**b**) upon compression (vanishing of LLPS) at a constant *p*-jump amplitude of 1000 bar. The absorption data are normalized to their maximum values (1.0).

Figure 8 exhibits the effect of TMAO concentration on the transition kinetics of the *γ*D-crystallin solution at a concentration of 50 mg mL^−1^ at *T* = 3 °C and a constant *p*-jump amplitude of 1000 bar (changing the *p*-jump amplitude has no significant effect on the kinetics of phase droplet formation, see Figure SI 4). Interestingly, the time constant for LLPS formation increases slightly with increasing TMAO-concentration: *t*_tr_ is 4.9 ± 0.1 s and 5.4 ± 0.1 s in 0.3 and 0.5 M TMAO upon LLPS formation at 3 °C (*t*_1/2 _≈ 2.2 s). This could be explained by the fact that with increasing TMAO concentration the formation of larger phase droplets is slightly disfavored as TMAO stabilizes the energetically costly formation of small droplets. TMAO owns the ability to interact favorably with bulk water, leading to an increase in hydrogen bonding and structuring of the solvent, and is preferentially excluded from the protein interface, thereby increasing its hydration. Owing to this excluded volume effect, more dense and compact structures are favored^25,26^, which leads to an increase of the surface tension and hence surface energy upon droplet formation. Therefore, the overall transition time for LLPS formation increases slightly (0.5 s) in the presence of TMAO. In the opposite direction, upon formation of the homogeneous phase, the kinetics is about a factor of 10 faster (*t*_tr _≈ 1.5 s, *t*_1/2 _≈ 0.1 s), as in the neat buffer solution, and no significant effect on the phase transition kinetics is recorded (Tables SI 2.4 and 2.6). Though the concentration of the cosolute TMAO in the dense droplet phase cannot be easily determined, owing to the fact that the TMAO preferentially interacts with bulk water and is preferentially excluded form protein interface, its concentration in the dense phase is expected to be much smaller, hence imposing a lesser effect on the dissolution kinetics, as observed here. Similar to the neat buffer data, an increase of temperature from 3 to 11 °C results in a slight shift (about 0.6 s in *t*_tr_ and 0.2 s in *t*_1/2_) to smaller time constants.

We have seen from the equilibrium measurements discussed above that urea has a marked effect on the stability of the LLPS, leading to vanishing of the LLPS at concentrations above 0.5 M urea. Figures SI 5c,d show the effect of different urea concentrations on the transition kinetics of the *γ*D-crystallin solution at a concentration of 50 mg mL^−1^ at *T* = − 2 °C and a constant *p*-jump amplitude of 1000 bar (changing the *p*-jump amplitude has no significant effect on the kinetics of phase droplet formation, Figures SI 5a,b). Different from the effect of urea at submolar concentrations on the equilibrium phase behavior, the addition of 0.2 M and 0.3 M urea has no significant effect on the phase transition kinetics of *γ*D-crystallin compared to the neat buffer solution.

**Figure 9:**
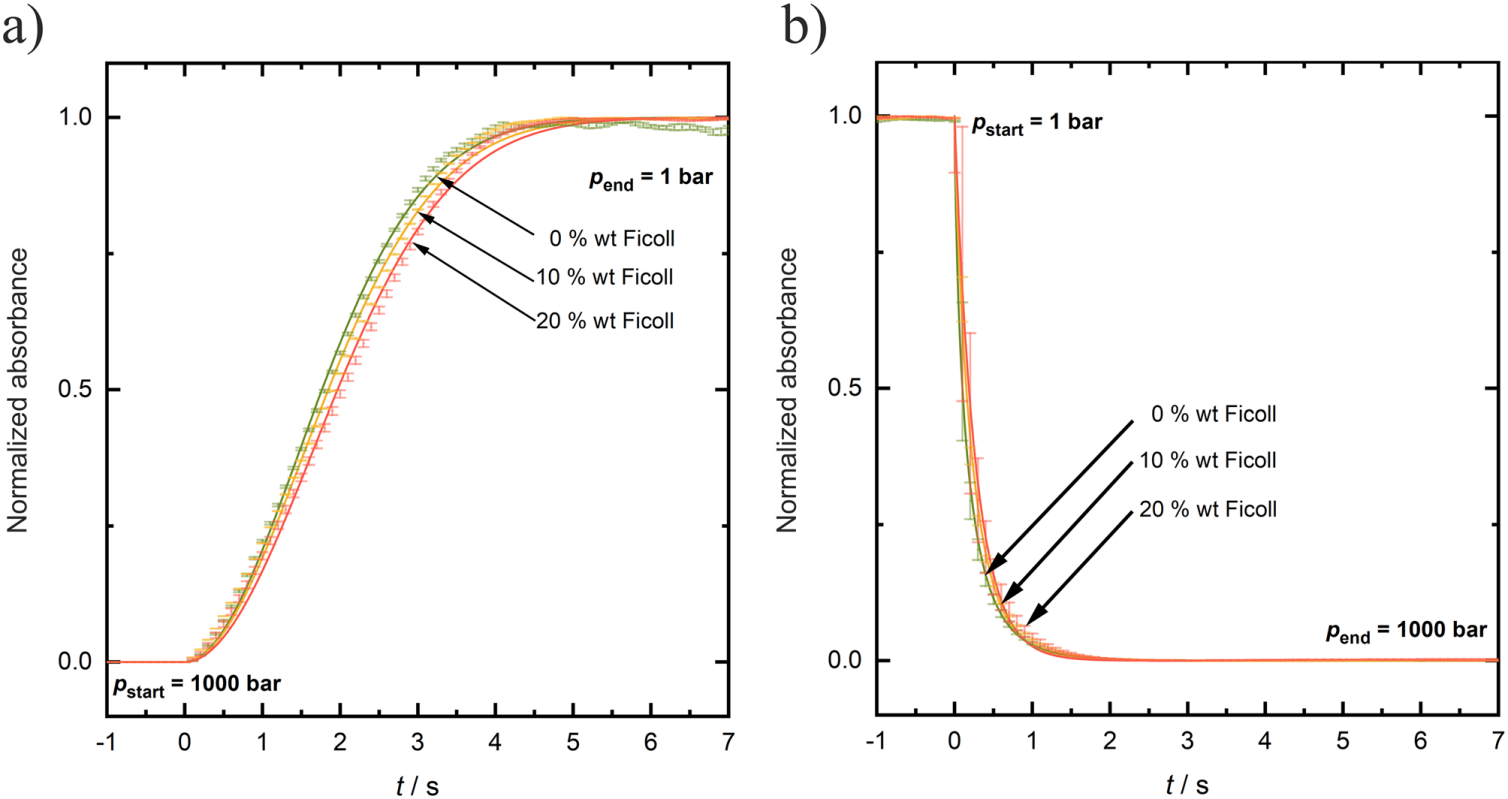
Effect of Ficoll concentration on the kinetics of LLPS formation of a 50 mg mL^−1^ *γ*D-crystallin solution at *T* = 3 °C (**a**) upon decompression (LLPS formation) and (**b**) upon compression (vanishing of LLPS) at a constant *p*-jump amplitude of 1000 bar. The absorption data are normalized to their maximum values (1.0).

Figure 9 depicts the effect of the macromolecular crowding agent Ficoll at different concentrations on the transition kinetics of the LLPS of *γ*D-crystallin at *T* = 3 °C and a constant *p*-jump amplitude of 1000 bar. Also in this case, changing the *p*-jump amplitude has no effect on the kinetics of phase droplet formation (Figure SI 6). The time constant for LLPS formation increases slightly with increasing Ficoll concentration: *t*_tr_ is 4.4 ± 0.1 s, 5.0 ± 0.1 s and 5.3 ± 0.1 s in 0, 10 wt% and 20 wt% Ficoll upon LLPS formation at 3 °C. Such behavior is similar to that seen for TMAO. The slight increase of the time constant for LLPS formation can be explained by the excluded volume effect imposed by the crowder, which is susceptible to stabilize smaller droplets, thereby reducing their growth rate. In the opposite direction, upon formation of the homogeneous phase, the kinetics is again faster, *t*_tr _≈ 1.5 s and *t*_1/2 _≈ 0.2 s, as in the neat buffer solution, and no significant effect on the phase transition kinetics is recorded (Tables SI 2.15 and 2.16), which may be expected as the macromolecular crowder is largely excluded from the droplet phase, which is also the reason for the droplet's increased temperature stability.

To quantify the kinetic data, the kinetic profiles were fit to the Johnson–Mehl–Avrami–Kolmogorov (JMAK) function (Eq. 2) to yield the Avrami exponent, *n*, which can take on physically plausible values between 1 and 4. The parameter *k,* a rate constant, depends on the nucleation and growth rate, which both depend also on temperature and may change with time. As can be seen from Tables SI 2.5, 2.9 and 2.15, the Avrami parameter *n* obtained is 1.9 ± 0.2 for all solution conditions upon LLPS formation, within the experimental error independent of the concentration of the cosolutes added. The kinetic constant, *k*, is on the order of 0.2 s^−1^. Whereas the rate constant in neat buffer and the urea solution are similar, the kinetic constants for LLPS formation decreases slightly upon addition of TMAO and Ficoll, e.g. from *k* = 0.24 s^−1^ to 0.15 s^−1^ in 0.5 M TMAO and to *k* = 0.17 s^−1^ in 20 wt% Ficoll at *T* = 3 °C, reflecting a decrease in nucleation and growth rate upon addition of these cosolutes.

In the opposite direction, upon formation of the homogeneous phase out of the LLPS region, the *n*-value decreases and takes on values around 1.0 (*n* = 0.9 ± 0.1) for all solution conditions. Here, no nucleation and growth processes are involved, the droplets rapidly dissolve and form a homogeneous phase, only. In accordance with the *t*_tr_ and *t*_1/2_ values reported above, the rate constant increases to about 3.5 ± 0.5 s^−1^ at *T* = 3 °C for the LLPS → homogeneous phase transition, and is similar for all solution conditions.

An Avrami parameter of *n *≈ 2 indicates that the nucleation rate upon LLPS formation is not constant, which would lead to *n* = 4 for homogeneous nucleation or *n* = 3 for the unlikely case of heterogeneous nucleation^11,36,37^. Here, a diffusion-limited growth mechanism is observed, which predicts values of *n* = 5/2 for a constant rate of homogeneous nucleation. The small deviation from *n* = 5/2 might be due to the fact that the growth rate changes with time. This is also reflected in small deviations between the fits and experimental data points at early and late times of the transition. A spinodal decomposition scenario was not observed for any solution condition and pressure-jump amplitude at these protein concentrations below 100 mg mL^−1^, indicating that transitions occurred only between the homogeneous phase and the metastable two-phase region (Fig. 1).

## Summary and conclusions

Using human γD-crystallin as model system for liquid–liquid phase separation phenomena of globular proteins, we have studied the effect of various cosolutes encountered under cell-like conditions on the equilibrium phase behavior of its two-phase coexistence region and how the phase transition kinetics depends on the cosolutes. To this end, the *p*-jump relaxation method has been employed, which allowed us to study the kinetics and its underlying mechanism under isothermal conditions, i.e., without changing the thermal energy and stability of the system.

As a prerequisite of the kinetic measurements, first, the stability of the protein has been studied for all solvent conditions, temperatures and pressures used, and the *p*,*T*,*c*-phase diagrams of the LLPS have been determined (Figs. 5, 6). Using the theory of critical point phenomena, the critical concentration and temperature of the pure protein system could be set at *c*_c _≈ 195 mg mL^−1^ and *T*_c _≈ 13 °C, respectively. No changes in secondary structure of the protein are observed upon droplet formation.

Generally, an increase of temperature and pressure disfavors LLPS formation of the protein. The phase-separated condensed phase of γD-crystallin at low temperature and ambient pressure is stabilized by transient electrostatic, hydrophobic and van der Waals interactions among neighboring protein molecules^9,10^. The dissociation of the droplet phase at high temperatures can be rationalized by an increase of the mixing entropy, which dominates over the cohesive intermolecular interaction term at sufficiently high temperatures. On the other hand, a large number of voids, i.e., water-free cavities, are created by the transiently touching protein molecules in the condensed liquid-like droplet state. Hence, the condensed droplet phase becomes unstable under high pressure since, according to Le Châtelier's principle^18^, a reduction of void volume is achievable by a homogeneous dilute phase, which is also favored by a higher mixing entropy^10^. Moreover, high pressure destabilizes hydrophobic contacts relative to compact yet solvent-separated configurations^10,38^.

The compatible cosolute TMAO and the crowding agent Ficoll revealed a drastic stabilizing effect of the LLPS region. Increases of the cloud point temperature, Δ*T*_cloud_, by about 12 °C and 10 °C upon addition of 0.3 M TMAO and 20 wt% Ficoll, respectively, were observed for a 50 mg mL^−1^ γD-crystallin solution. A corresponding marked increase of the pressure stability of the LLPS region has also been observed (e.g., Δ*p*_cloud _≈ 600 bar for 0.3 M TMAO and Δ*p*_cloud _≈ 600 bar for 15 wt% Ficoll). A plausible physical rationalization of the increase of the temperature and pressure stability of the LLPS of γD-crystallin by TMAO has been discussed before^10,12^. TMAO stabilizes compact protein configurations because it interacts strongly with water but unfavorably with proteins, as evident from the fact that TMAO is depleted around protein surfaces (so-called solvophobic effect)^25,26^. The presence of TMAO can therefore counteract droplet dissolution by pressure as the dense droplet phase has less solvent-exposed protein surface and therefore fewer unfavorable interactions with TMAO compared with the larger total solvent-exposed protein surface in the dispersed homogeneous phase. A similar scenario can be envisaged for the crowding agent Ficoll. Owing to the repulsive excluded volume effect imposed by the crowding agent, condensed structures are favored in such situation as well, which results in an increase of the temperature and pressure stability of the droplet phase of the protein. Of note, the drastic increase of the temperature and pressure stability of the LLPS region as shown here by addition of the deep-sea osmolyte TMAO and the macromolecular crowder opens up a window for organisms exposed to harsh environmental conditions to manipulate the stability of their protein condensates. Conversely, addition of the chaotrope urea destabilizes the LLPS. Such effect is most likely due to interaction of urea with the backbone and side chains of γD-crystallin, thereby reducing the weak intermolecular interactions between the protein molecules in the droplet phase. Remarkably, already 0.5 M urea is able to shift *T*_cloud_ below the freezing point of the solution.

Based on the phase diagram of the system determined for the various solution conditions, fast *p*-jumps across the two-phase boundaries were carried out to study the kinetics of the LLPS of γD-crystallin. As the pressure dependent processes were fully reversible, also bidirectional *p*-jumps could be carried out, which allowed us to determine the rates of formation and dissolution of the protein droplet phase. The formation of the droplet phase was found to be a very rapid process with overall transition times, *t*_tr_, of ~ 4 s, only. The time constant for LLPS formation increases slightly with increasing TMAO and macromolecular crowder concentration (Δ*t*_tr _≈ 1 s). The addition of 0.3 M urea has no significant effect on the phase transition kinetics. An increase of temperature results in a slight increase of the rate of the phase transition (e.g., Δ*t*_tr _≈ − 0.6 s from − 3 to 11 °C). In the opposite direction, upon formation of the homogeneous phase, i.e. upon dissolution of the protein droplets, the kinetics is about a factor of 10 faster (*t*_tr_ ≈ 1.5 s) and similar for all solution conditions.

To quantify the kinetics data, the kinetic profiles were fit to the Johnson–Mehl–Avrami–Kolmogorov (JMAK) model to yield the Avrami exponent, *n*, and the rate constant, *k*. Even at the highest protein concentrations used, no dynamic arrest or gel formation has been observed like in highly concentrated lysozyme and antibody solutions^7,8,39,40^. The Avrami parameter *n* is close to 2 for all solution conditions upon LLPS formation, and the kinetic constant, *k*, is on the order of 0.2 s^−1^. Whereas the rate constant in neat buffer and the urea solution are similar, the kinetic constants for the LLPS formation decreases slightly upon addition of TMAO and Ficoll, e.g. from *k* = 0.24 s^−1^ to 0.15 s^−1^ in 0.5 M TMAO and 0.17 s^−1^ in 20 wt% Ficoll, respectively, at *T* = 3 °C, reflecting a minor decrease in nucleation and growth rate upon addition of these cosolutes. Hence, upon addition of TMAO and Ficoll, the barrier to droplet formation and coalescence increases, leading to a retardation of the phase transition. Such small reduction on the kinetics of nucleation and growth rate would also be consistent with a general confinement effect. The fraction of droplets, in which nucleation has occurred, is given by *f* = 1 − exp[− *J*_v_ *N V*_d_] (*J*_v_ is the steady-state intrinsic nucleation rate, *N* and *V*_d_ is the number and volume of the confining space, respectively), this is to say, even if the barrier is unchanged, the nucleation rate *J* = *J*_v_ *N V*_d_ decreases with decreasing system size^41^.

An Avrami parameter of *n *≈ 2 indicates that the nucleation rate upon LLPS formation is not constant. Rather, the phase transition seems to proceed via a diffusion-limited nucleation and growth mechanism at these subcritical protein concentrations, with the growth rate probably changing with time. Such scenario is also expected to be the case within biologically relevant (e.g., intracellular) crowded systems. A spinodal decomposition scenario was not observed for any solution condition and pressure-jump amplitude, indicating that transitions occurred only between the homogeneous phase and the metastable two-phase region.

Hence, overall, formation and dissolution of the protein condensate is a very rapid process taking place on the seconds timescale, i.e. it occurs on a biologically relevant short time period. The rapid kinetics of formation or dissolution of a particular LLPS is expected to meet the pertinent timescale required for the biochemical processes taking place in such assemblies, such as stress-dependent sequestration of signaling molecules forming signaling clusters or the co-localization of biomolecular units needed for enhancement in reactivity via co-localization of enzymes for substrate channeling. Despite the marked effect cell-like cosolutes take on the stability of the LLPS region, their effect at biologically relevant concentrations on the phase transformation kinetics is almost negligible, which might be a particular advantage in the cellular context, as a rapid kinetics of LLPS formation, i.e. a fast switchability of the transition, should not be compromised by the presence of cosolutes. Of note, organisms thriving under high-pressure conditions in the deep sea, with pressures of up to 1 kbar, have to cope with the pressure sensitivity of biomolecular condensates to avoid detrimental impacts to their cell physiology^12^. Our experiments demonstrate that the deep-sea cosolute TMAO, an osmolyte upregulated in deep-sea fish, significantly enhances the stability of the condensed protein droplets, but does not significantly affect the rapid kinetics of LLPS formation and dissolution of globular protein condensates.

## Experimental procedures

### FT-IR spectroscopy

The pressure-dependent FTIR measurements were performed using a Nicolet 6700 FTIR spectrometer (Thermo Fisher Scientific, Waltham, MA, USA) equipped with a nitrogen-cooled MCT detector and a diamond anvil cell with Type IIa-diamonds having a surface diameter of 0.6 mm^20^. Stainless steel with a thickness of 50 µm and a central hole with a diameter of 0.45 mm was used as spacer between the two diamonds. The resulting sample volume was 10 nL. The temperature of the diamond anvil cell was regulated by an external water bath. In addition, the entire measuring chamber was continuously flushed with dry air to reduce the amount of water vapor in the beam path and thereby to minimize signal noise. As pressure indicator barium sulfate (BaSO_4_) was used. BaSO_4_ shows a pressure-sensitive vibration of the sulphate group at 983 cm^−1^, whose band position shifts to higher wave numbers with increasing pressure^42^. The measurable pressure tolerance is approx. ± 200 bar. For all measurements the spectral resolution was 2 cm^−1^. The apodization was performed by a Happ–Genzel function. Before each measuring series, the spectrometer was calibrated by using the OMNIC software (Thermo Fisher Scientific, Waltham, MA, USA). Subsequently the obtained data were processed with the software GRAMS/AI 8 (Thermo Fisher Scientific, Waltham, MA, USA). To this end, the respective buffer spectrum was subtracted from the sample spectrum and the amide I′ band region (1600–1700 cm^−1^) was analyzed. After baseline correction, the amide I′ band was normalized. In the first step of the secondary structure analysis, the maxima of the subbands were determined. For this purpose, the FSD treated spectra were compared with the second derivative of the spectra. Afterwards the number, position and half-width of the subbands were determined. Then, the number, position and half-width of the subbands were determined. The fitting of the subbands to the amide I′ band was done with mixed Gaussian-Lorentz functions. The variation of each subband position was limited to ± 2 cm^−1^ and the area of each subband corresponds to the percentage of the total secondary structure of the protein.

A ~ 5 wt% (50 mg mL^−1^) *γ*D-crystallin solution was prepared using D_2_O instead of H_2_O as solvent because H_2_O has a strong absorption band at 1645 cm^−1^ and therefore absorbs in the protein conformation-sensitive amide I′ band region. Owing to its pressure-stability, a Tris-D_2_O buffer with pD = 7.4 (pD = pH meter display + 0.4) was used.

### High-pressure microscopy

For the pressure dependent microscopy experiments, a home-built high-pressure cell was used^9,10,12^. Temperature was controlled by a circulating water bath. Pressure was generated hydrostatically by a high-pressure hand pump with water as pressure-transmitting fluid. Flat diamond windows of 0.8 mm thickness were used as optical window material on both sides. The design fits to a standard inverted microscope with off-the-shelf long-distance microscope objectives. Details are given elsewhere^9,10,12^.

## Turbidimetry (UV/Vis absorption) measurements

### Experimental

The temperature dependent UV/Vis measurements were performed using a Shimadzu UV-1800, with 2 nm resolution in the wavelength range 250–550 nm. LLPS was examined by monitoring the turbidity (apparent absorption) through light scattering at 400 nm. The temperature of the sample cell was controlled by an external water thermostat. The pressure dependent measurements were carried out on a PerkinElmer Lambda 25 spectrophotometer with a home-built high-pressure optical cell. As window material, sapphires with a diameter of 20 mm and a thickness of 10 mm were used, which enclosed the sample chamber on both sides via O-ring seals^9,10,12^. The layer thickness of the sample was about 1 mm. Pressure was applied using a high-pressure hand pump from Nova SWISS and was measured by a pressure sensor (Burster Präzisionsmesstechnik, Gernsbach). The pressurizing medium was water. The temperature was controlled by an external water bath and the measuring chamber was flushed with dry air during the entire measurement to avoid condensation of water droplets on the windows at low temperatures.

Pressure-jumps were facilitated by opening of an air-operated valve between the high-pressure cell and a liquid reservoir container. With the pressure-jump apparatus (deadtime ~ 50–100 ms) variable amplitude pressure-jumps (up to 1.6 kbar) in both directions were possible. To minimize the effect of an adiabatic temperature change in the course of the pressure-jump, the high-pressure cell was constructed to hold only a very small volume of the pressurizing medium. To ensure equilibrium of the system before and after the pressure jump, the total measuring time of each experiment was about 100 s. The resulting pressure-jump data were analyzed using Origin 2019 (OriginLab Corporation, Northampton, USA). An absorption (turbidity) of *A* = 0 indicates a homogeneous clear solution. The transition time reported results from the starting point at *t* = 0 and the time *t* at which the turbidity curve intersects the baseline (*A* = 0) or maximum value (*A* = 1), respectively. The half-life time, *t*_1/2_, is the time where half of the absorption intensity changes is reached.

### Theoretical considerations

Precise determination of liquid–liquid phase boundaries is prerequisite for studying the kinetics of phase transitions. The formation of liquid droplets upon entering the phase-separated system leads to an increase of the turbidity of the liquid mixture. Therefore, optical light scattering methods can be used to observe the phase transition^11,36,43,44^. The kinetics of phase separation can vary greatly with time, depending on the system and the area of the phase diagram (see Fig. 1)^36^. Phase separation in the metastable region occurs through a process of nucleation and growth, in which tiny droplets of the new phase are formed through thermal, density, and compositional fluctuations. Following nucleation, the droplets grow through diffusion of the solute molecules and coalesce. However, the nucleation rate is hampered by an energy barrier associated with the formation of the droplet interface. Hence, nucleation is only successful when the droplets reach a critical size, so that the unfavorable interfacial energy cost is exceeded by the volume energy recovery. The subsequent growth of the droplets is then limited by molecular diffusion. Conversely, quenching near the critical point region (CP) and below the spinodal line causes phase separation through a different process, denoted as spinodal decomposition^45^. In this case, domains of high and low concentrations form spontaneously throughout the entire liquid. In our case, the first mechanism, i.e., nucleation and growth, is observed and will hence be considered in the following, only. The measured turbidity, *τ*, of the sample, changes when the cloud point is reached, which is indictive of phase separation and marks the onset of phase separation. which in turn reduces the initial light intensity, *I*_0_, of the light transmitted through the sample, $I = I_{0} \exp ( - \tau l)$, with l being the optical path length of the sample^11^. The turbidity of the sample includes contributions from thermal and density fluctuations of the homogeneous bulk solution and a generally much larger contribution from the droplets formed. The latter is proportional to the number density of droplets and their scattering cross section, which depends markedly on the size of the droplets. For the initial small droplet radii, *r*, the scattering signal can be described by the Rayleigh theory^11,43^, leading to a turbidity which is proportional to *r*^6^. For large droplet radii, the scattering cross section rather follows the Rayleigh–Gans–Debye or Mie theory and is proportional to *r*^4^^11,43^. Next to the droplet number density formed from a jump into the two-phase region, the transmitted light attenuation will also depend on various other factors, such as the droplet size distribution, the optical path length, and stirring.

Approaching the critical point region, light scattering increases dramatically owing to the divergence of the correlation lengths of density and concentration fluctuations, *ξ* ∝ [(*T*_c_ − *T*)/*T*_c_]^−2*ν*^, with critical exponent *ν* = 0.63, finally approaching visible light dimensions and leading to critical opalescence^34,35,37^. Being far off the critical point and spinodal phase region in our case, such scenario is not observed here. The turbidity or absorption, *A* = log(*I*_o_/*I*), recorded here is controlled by droplet scattering only, and depends largely on the number of phase droplets formed and their size distribution, i.e. is a sensitive measure of the volume fraction of the droplet phase formed. In general thermodynamic terms, turbidity (and the forward scattering angle Rayleigh ratio) is linked to the Hessian matrix of second derivatives of the Gibbs free energy of the solution per unit volume with respect to the number densities of the components, which determines the stability of the solution with respect to phase separation^46^.

### Nucleation and growth models

Treatment of the experimental kinetic data using theoretical approaches are expected to contribute to the fundamental understanding of LLPS phenomena and the underlying key molecular-scale driving forces of the protein assembly, as well as of droplet phase transitions in intracellular organization in general.

If the phase boundary is crossed away from a critical point and phase separation begins between the binodal and spinodal, as in our case, then a first order transition occurs. Here, nucleation and growth of droplets of the new phase emerges, and then coarsens toward the finally demixed equilibrium state. The phase transition kinetics can be divided into nucleation growth processes, which typically dominate during the early stages, and coarsening processes (e.g., Ostwald ripening) which dominate during the late stages of transformation. One commonly observed consequence is that the droplet size and its distribution, i.e., the average domain or droplet size $\left\langle R \right\rangle$, increases with a power law dependence on time, e.g. $\left\langle R \right\rangle$ = *K t*^*m*^^36^. Comparing measurements of quantities such as *R* with theoretically-determined steady-state exponents *m* and prefactors *K* can often help to identify the specific growth or coarsening mechanisms at work. An alternative kinetic analysis, which we followed, can be performed by measuring the total volume fraction of transformed droplet phase (d), *θ*_d_(*t*) = *V*_d_(*t*)/*V*, as a function of time. Such data can be compared with theories aimed at predicting the evolution of *θ*_d_ according to the type of nucleation and growth kinetics in operation. One approach, the phenomenological Johnson–Mehl–Avrami–Kolmogorov (JMAK) theory, has been widely employed to analyze the kinetics of first-order phase transformations^36,47–50^. Under the typical assumption of random nucleation and uniform growth rates, *θ*_d_ is predicted by JMAK-type theory to evolve according to

2 $$\theta_{{\mathrm{d}}} = \frac{{V_{{\mathrm{d}}} (t)}}{V} = 1 - C \exp ( - kt^{n} )$$

when entering the two-phase region, where *C* and* k* are constants, and *n* is the so-called Avrami exponent. Values for *n* can be extracted from fits to *θ*_d_(*t*). Physically plausible mechanisms give Avrami exponents between 1 and 4. The values of *k* and *n* can be obtained from experimental data by plotting ln[− ln(1 − $\theta_{{\mathrm{d}}}$)] vs. ln[*t*]. A commonly encountered case is that of growth at a constant rate, i.e. $\left\langle {R(r)} \right\rangle$ ~ *t* or volume of transferred phase *V*(*t*) ~ *t*^3^, which yields *n* = 4 for homogeneous nucleation (i.e., nucleation rate, *J* (*t*) = constant) and *k* = (π/3)*G*^3^*I*_*V*_ (*I*_*V*_ = nucleation rate density, *G* = growth rate of the spherical particles)*.* For heterogeneous nucleation (e.g., nucleation at interfaces), *n* = 3^11,36,37^.

Assumptions employed by the JMAK theory are violated when diffusion controls domain growth rates, as may also be the case within many biologically relevant (e.g., intracellular) crowded systems. Such situations will most likely take place at later growth stages when the diffusion zones of different droplets start to overlap. In this regime of diffusion limited growth (DLG), JMAK theory predicts *n* = 5/2 for a constant rate of homogeneous nucleation, and *n* = 3/2 for heterogeneous nucleation^11^.

Depending on the particular system under study, later stages of growth (coarsening), when approaching the plateau in *θ*_*d*_ versus *t*, may require more sophistical approaches and another theory in the cases of diffusion-limited growth and/or low volume fraction transformations, e.g. for diffusion-limited precipitation. At conditions when the diffusion zones of separated droplets overlap significantly, the approach to a steady-state *θ*_*d*_ value is characterized by *n *≈ 1. This effective late time exponent results from the gradual depletion of available excess solute. For example, it has been found that in vivo experimental data of optogenetically controlled FUS-based droplets do indeed exhibit the predicted linear *n* = 1 behavior during this regime^36,51^. An initial *n *≈ 2 nucleation/growth regime consistent with mixed heterogeneous (*n* = 3/2) and homogeneous (*n* = 5/2) nucleation and DLG is followed by a crossover to a second *n *≈ 1 regime, quantitatively consistent with that predicted by the theory of diffusion-limited precipitation when diffusion zones of nearby droplets begin to overlap.

## Supplementary information

Supplementary Information.
