# Author Correction: The effects of cosolutes and crowding on the kinetics of protein condensate formation based on liquid–liquid phase separation: a pressure-jump relaxation study

**DOI:** 10.1038/s41598-026-37234-2

**Published:** 2026-03-03

**Authors:** Hasan Cinar, Roland Winter

**Affiliations:** Physical Chemistry I, Biophysical Chemistry, Faculty of Chemistry and Chemical Biology, TU Dortmund University, Otto-Hahn-Strasse 4a, 44227 Dortmund, Germany

Correction to: *Scientific Reports* 10.1038/s41598-020-74271-x, published online 14 October 2020

The original version of this Article contained errors.

In the “Experimental Procedures” section under the subheading "Turbidimetry (UV/Vis absorption) measurements," a significant portion of the text and equations in the “Theoretical considerations” part was reproduced from Reference 35 and its supporting information without appropriate citation. The authors apologize for this oversight and have now rewritten the entire “Theoretical considerations” section to ensure original phrasing, while preserving the underlying scientific content. As a result,

“**Theoretical considerations.** Precise determination of liquid–liquid phase boundaries is prerequisite for the studying the kinetics of the phase transition. The formation of droplets when entering the phase-separated system causes a binary liquid mixture to become turbid. Consequently, light scattering optical techniques are commonly employed to observe the phase transitions ^35,43,44^. The temperature at which turbidity can first be identified, is referred to as the cloud point. The overall phase separation kinetics may vary widely in time, depending on the region of the phase diagram crossed and across different systems ^36^. A single-phase system is completely unstable within the spinodal curve of the two-phase system, and metastable in the regions between the coexistence curve (the binodal) and the spinodal curve (Fig. 1). The phase separation processes are different in these two regions. Phase separation in the metastable region occurs through a process of nucleation and growth, where, through instantaneous thermal and compositional fluctuations, tiny droplets of the new phase form, grow by solute diffusion, and combine. The rate of nucleation is hindered by an energy barrier associated with forming the boundary surface of the new phase. Nucleation is successful if thermal fluctuations allow the droplet to reach a critical size such that the surface energy costs are exceeded by the volume energy returns. Following the creation of the droplet, the local surroundings will be depleted of solute, and subsequent droplet growth is limited by molecular diffusion along this concentration gradient.

In contrast to jumps into the metastable region, for quenches near the critical point region and below the spinodal, phase separation takes place by a different process known as spinodal decomposition ^45^. Here, domains of higher and lower concentrations develop spontaneously across the entire fluid all at once. In our case, the first mechanism, nucleation and growth, is observed, only, and will hence be considered in the following, only.

Induction of phase separation increases the turbidity, $\tau$, of the sample, which in turn decreases the intensity (or absorbance) $\tau$ of light transmitted through the sample ^35^,

2 $$I = I_{0} {\mathrm{exp}}( - t l)$$

where *I*_0_ is the incident light intensity and *l* is the optical path length. The total sample turbidity includes contributions from the homogeneous bulk solution as well as from the droplets once they have formed:

3 $$t{\mathrm{total}} = t{\mathrm{droplet}} + t{\mathrm{bulk}}$$

The temperature at which a discontinuity in turbidity occurs due to droplet formation, the cloud point temperature, is assumed to coincide with the coexistence curve temperature. Here we briefly discuss the theoretical predictions for both droplet and bulk scattering contribution. If no multiple scattering takes place, then the turbidity for scattering by droplets is

4 $$t{\mathrm{droplet}} = N C$$

where *N* is the droplet number density and *C* is the droplet scattering cross section, which depends on the size of the droplets (see below). The value of *C* may increase several orders of magnitude when the droplet radius increases, hence the light scattering signal changes abruptly at the coexistence curve temperature when *T*droplet suddenly becomes nonzero.

The second contributor to Eq. (3), bulk, arises from spontaneous composition and density fluctuations in the bulk liquid solution. Once droplets form, droplet is usually much greater than the baseline turbidity from the bulk. However, concentration fluctuations become very large on the spinodal curve and at in the critical point region (critical opalescence). In our case, we are remote from the critical point region.

For small droplet radii, when *x* <  < 1, where *x* = *k* *a* = 2π*a*/*λ* (*k* = 2π/*λ* modulus of the wave vector* r* = radius of the particle, *λ* wavelength of light, 400 nm), the scattering signal (for linearly polarized light) can be described by the Rayleigh theory ^35,43^:

5 $${I}_{\mathrm{R}}={I}_{0}\frac{4{k}^{4}{r}^{6}}{9{R}^{2}}{(m-1)}^{2}$$

where *R* is the distance to the detector and *m* = *n*_d_/*n*_bulk_ the ratio of the refractive index of the droplet, d, to the refractive index of the bulk medium (typically, 0.9 < *m* < 1.1). For Rayleigh scattering, the turbidity is given by

6 $${C}_{\mathrm{R}}\approx\uppi {k}^{4}{r}^{6}{(m-1)}^{2}$$

Once droplet radius exceeds *λ*/10, the light scattering signal is better described by Rayleigh-Gans-Debye (RGD) theory of by the Mie theory for larger droplets. The RGD scattering cross section is ^35^:

7 $${C}_{\mathrm{R}}\approx 2\uppi {k}^{2}{r}^{4}{(m-1)}^{2}$$

Hence, as the droplet radius undergoes a two order of magnitude increase from 10 nm to 1 µm, the scattering cross section increases by ten orders of magnitude.

The resulting increase in turbidity due to droplet formation after a jump into the two-phase region can then be calculated with Eq. 4. Next to the droplet number density, *N,* formed from a particular jump into the two-phase region, transmitted light attenuation will also depend on various other factors, such as the droplet size distribution, the optical path length, and stirring.

Approaching the critical point region, light scattering increases dramatically owing to the divergence of the correlation lengths of density and concentration fluctuations, *ξ*∞[(*T*_c_-*T*)/*T*_c_]^-2*ν*^, with critical exponent *ν* = 0.63, finally approaching visible light dimensions and leading to critical opalescence ^33,34,37^. An additional light scattering phenomenon occurs also when an unstirred critical solution undergoes spinodal decomposition below the spinodal line, where concentration gradients occur everywhere in the solution that grow in size with time. Light incident on a solution in the midst of spinodal decomposition scatters in a characteristic ring pattern similar to X-ray diffraction powder patterns. As the domains grow in size over time, the ring intensity increases and the ring diameter collapses ^35^. Being far off the critical point and spinodal phase region in our case, such scenario is not observed here. The turbidity or absorption, *A* = log(*I*_o_/*I*), recorded here is controlled by droplet scattering only, and depends largely on the number of phase droplets formed and their size distribution (Eqs. 5–7), i.e. is a sensitive measure of the volume fraction of the droplet phase formed. In general thermodynamic terms, turbidity (and the forward scattering angle Rayleigh ratio) is linked to the Hessian matrix of second derivatives of the Gibbs free energy of the solution per unit volume with respect to the number densities of the components, which determines the stability of the solution with respect to phase separation ^46^.”

now reads:

“**Theoretical considerations.** Precise determination of liquid–liquid phase boundaries is prerequisite for studying the kinetics of phase transitions. The formation of liquid droplets upon entering the phase-separated system leads to an increase of the turbidity of the liquid mixture. Therefore, optical light scattering methods can be used to observe the phase transition ^35,36,43,44^. The kinetics of phase separation can vary greatly with time, depending on the system and the area of the phase diagram (see Fig. 1) ^36^. Phase separation in the metastable region occurs through a process of nucleation and growth, in which tiny droplets of the new phase are formed through thermal, density, and compositional fluctuations. Following nucleation, the droplets grow through diffusion of the solute molecules and coalesce. However, the nucleation rate is hampered by an energy barrier associated with the formation of the droplet interface. Hence, nucleation is only successful when the droplets reach a critical size, so that the unfavorable interfacial energy cost is exceeded by the volume energy recovery. The subsequent growth of the droplets is then limited by molecular diffusion. Conversely, quenching near the critical point region (CP) and below the spinodal line causes phase separation through a different process, denoted as spinodal decomposition ^45^. In this case, domains of high and low concentrations form spontaneously throughout the entire liquid. In our case, the first mechanism, i.e., nucleation and growth, is observed and will hence be considered in the following, only. The measured turbidity, , of the sample, changes when the cloud point is reached, which is indictive of phase separation and marks the onset of phase separation. which in turn reduces the initial light intensity, $I = I_{0} {\mathrm{exp}}( - ~t~l)$ of the light transmitted through the sample, *I* = *I*_0_ exp(- ), with *l* being the optical path length of the sample ^35^. The turbidity of the sample includes contributions from thermal and density fluctuations of the homogeneous bulk solution and a generally much larger contribution from the droplets formed. The latter is proportional to the number density of droplets and their scattering cross section, which depends markedly on the size of the droplets. For the initial small droplet radii, *r*, the scattering signal can be described by the Rayleigh theory ^35,43^, leading to a turbidity which is proportional to *r*^6^. For large droplet radii, the scattering cross section rather follows the Rayleigh-Gans-Debye or Mie theory and is proportional to *r*^4 35,43^. Next to the droplet number density formed from a jump into the two-phase region, the transmitted light attenuation will also depend on various other factors, such as the droplet size distribution, the optical path length, and stirring.

Approaching the critical point region, light scattering increases dramatically owing to the divergence of the correlation lengths of density and concentration fluctuations, $\xi \infty \left[ {\left( {T_{{\mathrm{c}}} - T} \right)/T_{{\mathrm{c}}} } \right]^{{ - {\mathrm{2}}\nu }}$, with critical exponent *ν* = 0.63, finally approaching visible light dimensions and leading to critical opalescence ^33,34,37^. Being far off the critical point and spinodal phase region in our case, such scenario is not observed here. The turbidity or absorption, *A* = log(*I*_o_/*I*), recorded here is controlled by droplet scattering only, and depends largely on the number of phase droplets formed and their size distribution, i.e. is a sensitive measure of the volume fraction of the droplet phase formed. In general thermodynamic terms, turbidity (and the forward scattering angle Rayleigh ratio) is linked to the Hessian matrix of second derivatives of the Gibbs free energy of the solution per unit volume with respect to the number densities of the components, which determines the stability of the solution with respect to phase separation ^46^.”

Due to the deletion of the equations in the “Theoretical considerations”, all subsequent equations have been renumbered, and their corresponding cross-references have been updated.

Additionally, the first sentence of the legend for Figure 1 and the diagram in Figure 1a were reproduced from Reference 35 without attribution. The sentence has now been rephrased, and the diagram is now explicitly cited as being adapted from References 35.

The original Article has been corrected.

