# Supplementary material for: The effects of cosolutes and crowding on the kinetics of protein condensate formation based on liquid–liquid phase separation: a pressure-jump relaxation study
Source: Sci Rep. 2020 Oct 14;10:17245. doi: 10.1038/s41598-020-74271-x (PMC7566631; doi:10.1038/s41598-020-74271-x)
Supplement: Supplementary file 1 — Supplementary Information. [file 41598_2020_74271_MOESM1_ESM.docx]

**The Effects of Cosolutes and Crowding on the Kinetics of Protein Condensate Formation based on Liquid-Liquid Phase Separation - A Pressure-Jump Relaxation Study**

Hasan Cinar^1^ and Roland Winter^1^*

Physical Chemistry I - Biophysical Chemistry, Faculty of Chemistry and Chemical Biology,

TU Dortmund University, Otto-Hahn-Strasse 4a, 44227 Dortmund, Germany

* E-mail: roland.winter@tu-dortmund.de

**Supplementary Information**

**Additional Figures, Tables and Data**

e)


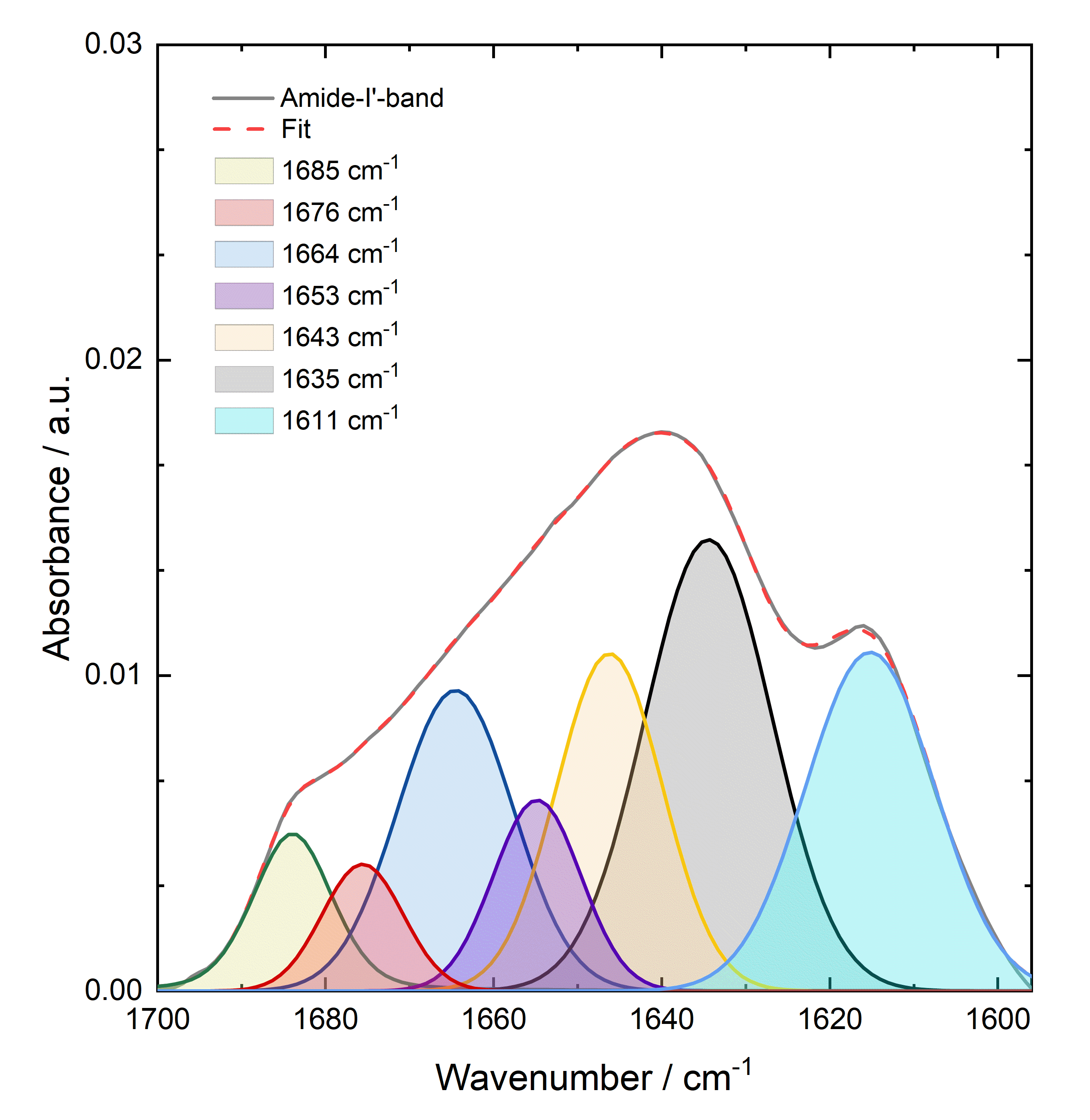


**Figure SI 1.** a) Normalized FTIR spectra of the pressure-dependent measurements on *γ*D-crystallin in the pressure range 1-10000 bar and 25 °C. b) Difference spectra where the 1 bar data have been subtracted from the spectra recorded at pressures between 1 and 10000 bar. c) Normalized FT-IR spectra of the pressure-dependent measurements on *γ*D-crystallin in the pressure range 1-10000 bar and 4 °C, and d) the corresponding difference spectra between 1 and 10000 bar. e) FTIR spectrum and deconvolution into subbands of *γ*D-crystallin at 80 ^o^C and ambient pressure after unfolding and subsequent aggregation (for band assignment, see also P. Evans, O. A. Bateman, C. Slingsby, B. A. Wallace, *Exp. Eye Res.* 84, 1001–1008 (2007).

Figure SI 1a shows the FT-IR absorption spectra of *γ*D-crystallin as a function of pressure at 25 °C. The amide I' band has a maximum at 1639 cm^-1^ at 1 bar, whose intensity decreases with increasing pressure. With increasing pressure, a shoulder forms at ~1620 cm^-1^, indicating a minor pressure-dependent structural change. This change is better visualized in the difference spectra shown in Figure 1b. The shift in intensity suggests that the fraction of disordered structures decreases with increasing pressure, while the fraction of a β*-*sheets/exposed side chains increases concomitantly. When comparing the pressure-dependent FT-IR absorption spectra of γD-crystallin at 4 °C with that at 25 ^o^C (Figures 1c,d), no significant differences can be seen. Figure SI 1e shows the FTIR spectrum and deconvolution into subbands of *γ*D-crystallin at 80 ^o^C and ambient pressure after unfolding (subband at ~1643 cm^-1^) and subsequent aggregation (subbands of intermolecular β-sheets at ~1611 cm^-1^ and ~1685 cm^-1^).

**Table SI 1**. Secondary structure elements of *γ*D-crystallin in solution at 4 and 24 °C (*p* = 1 bar).

| Wavenumber / cm^-1^ | 1685 ± 2 | 1676 ± 2 | 1664 ± 2 | 1653 ± 2 | 1643 ± 2 | 1635 ± 2 | 1611± 2 |
| --- | --- | --- | --- | --- | --- | --- | --- |
| Fractional band area (24°C) | 4.2% | 3.2% | 13.7% | 8.4% | 26.8% | 40.3% | 3.5% |
| Fractional band area (4°C) | 3% | 3.8% | 14% | 8.3% | 26.5% | 40.7% | 3.9% |


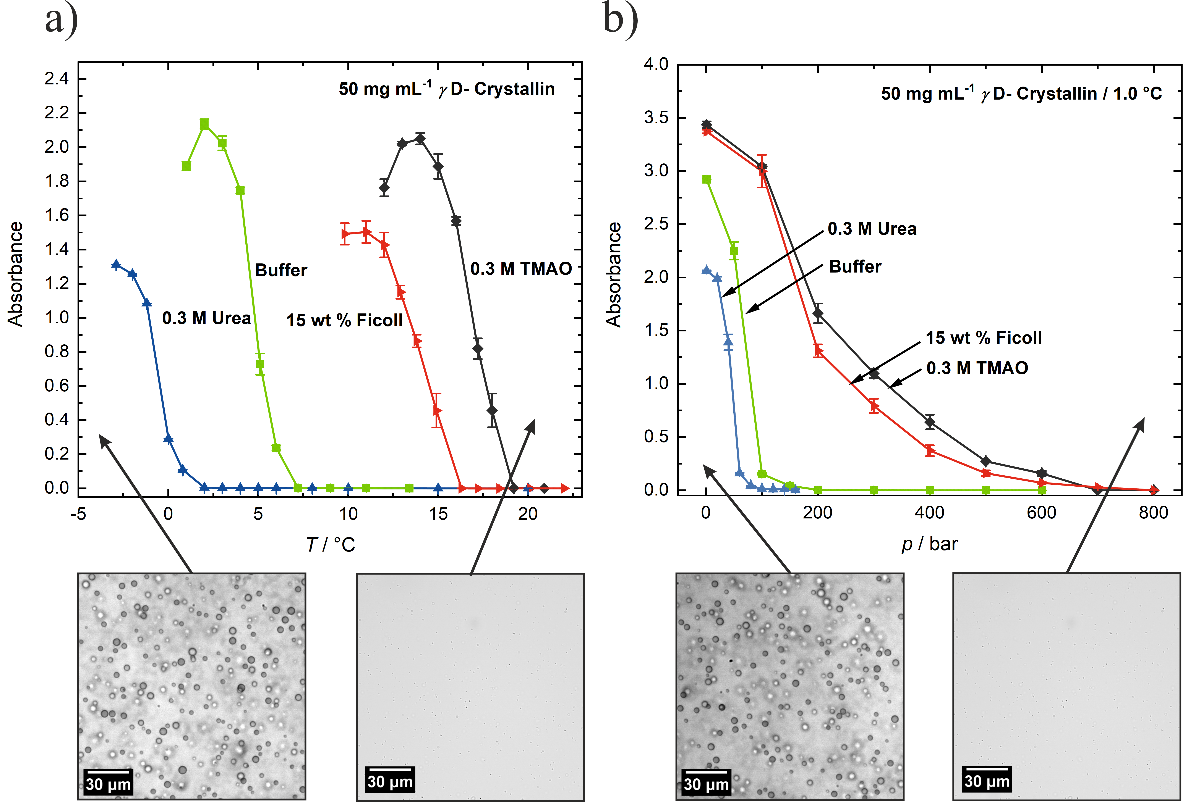


**Figure SI 2**. Representative UV/Vis absorption (turbidity) data (not normalized) at 400 nm of a 50 mg mL^-1^ solution of *γ*D-crystallin as a function of a) temperature in buffer (50 mM TRIS, 150 mM NaCl, pH 7.4), 0.3 M urea, 15 wt% Ficoll and 0.3 M TMAO, and b) pressure at *T* = 1 °C. Bottom: light microscopy snapshots of *γ*D-crystallin representing the phase-separated and the homogeneous state of the solution.


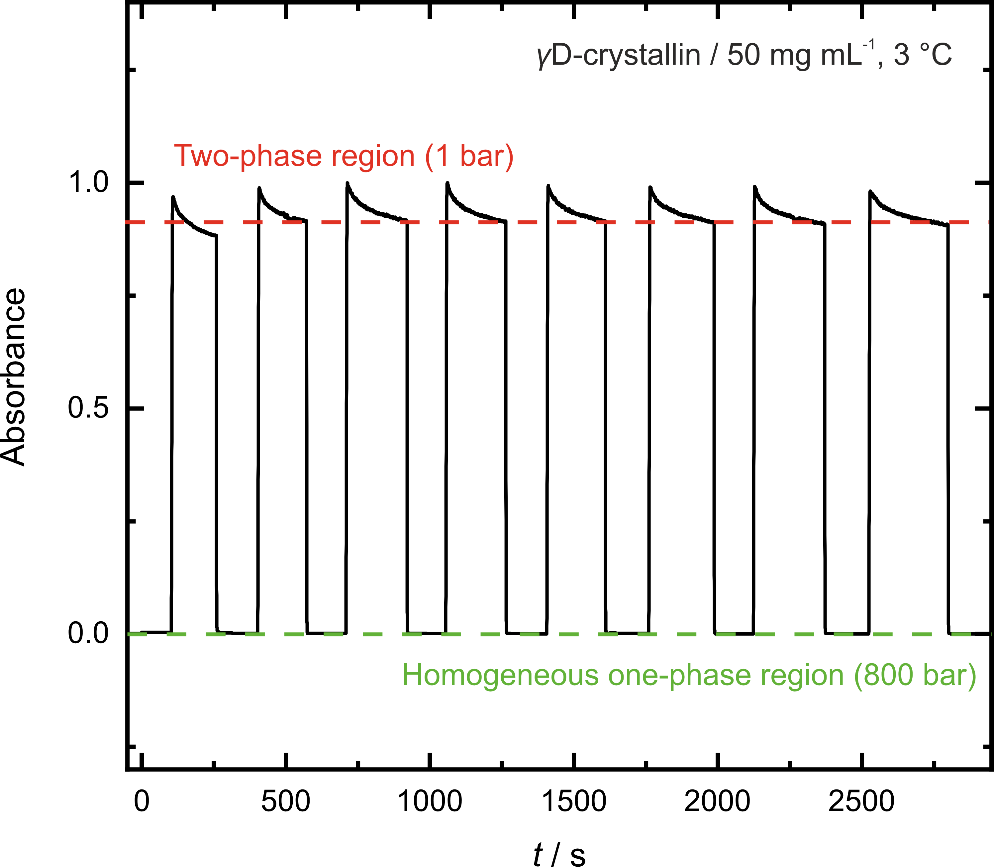


**Figure SI 3.** Pressure cycles across the LLPS phase transition region of *γ*D-crystallin in buffer solution at *T* = 3 ^o^C demonstrating the full reversibility of the transition.

**
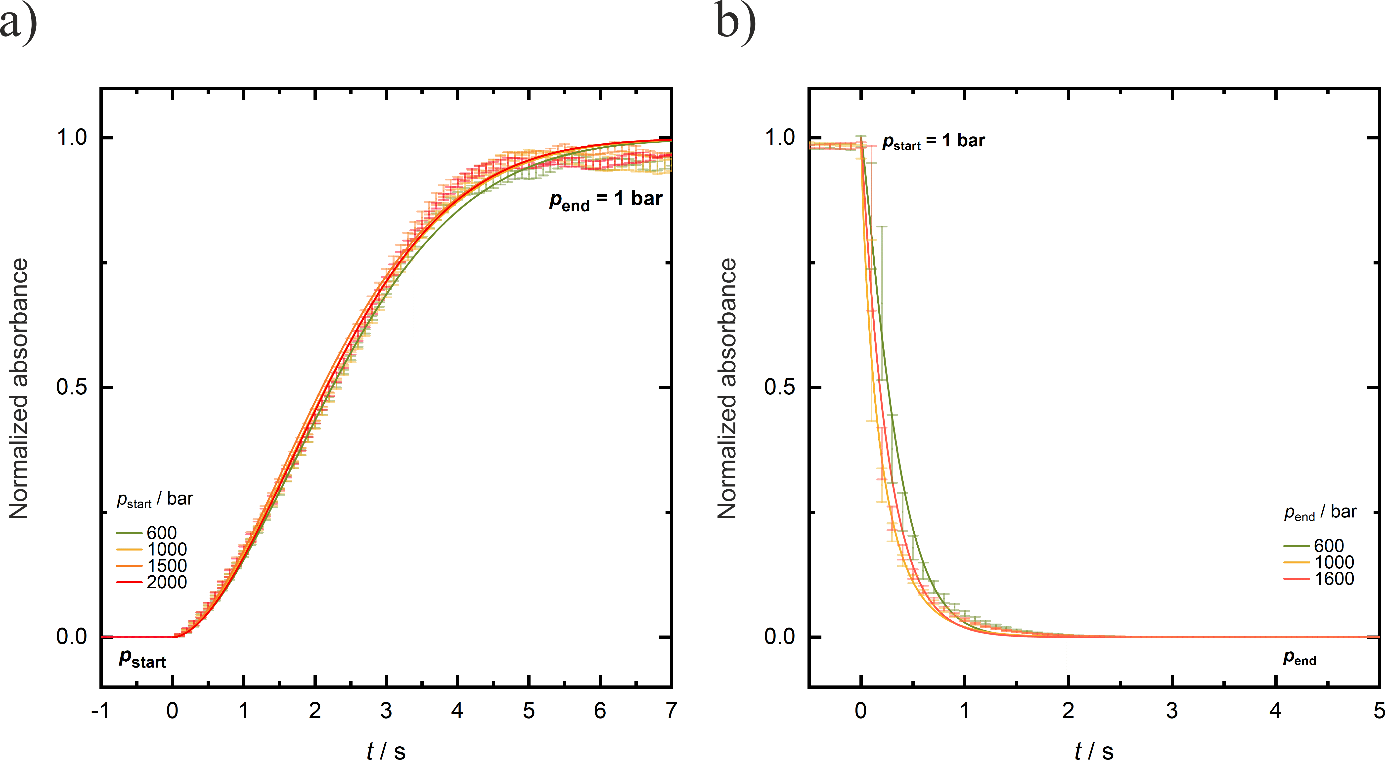
**

**Figure SI 4.** Effect of the pressure-jump amplitude on the LLPS phase transition kinetics of *γ*D-crystallin at a concentration of 50 mg mL^-1^ at 3 °C in the presence of 0.3 M TMAO upon a) pressure decrease and b) pressure increase. The absorption data are normalized to their maximum values (1.0).


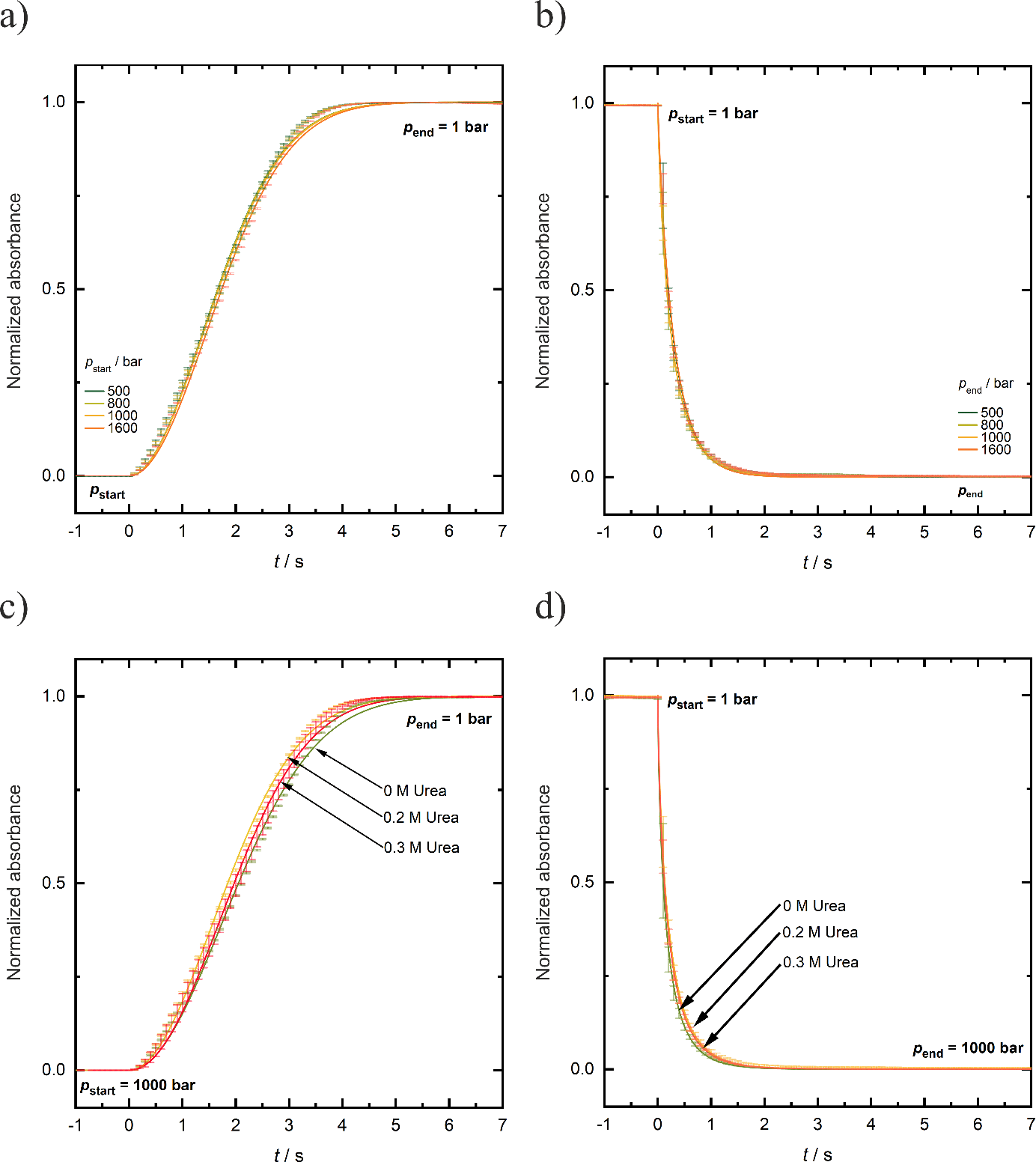


**Figure SI 5**. a, b): Effect of 0.2 M urea on the kinetics of LLPS of a 50 mg mL^-1^ *γ*D-crystallin solution at 2 °C a) upon decompression (LLPS formation) and b) upon compression (vanishing of LLPS) at different *p*-jump amplitudes. c, d): Effect of urea concentration on the kinetics of LLPS formation of a 50 mg mL^-1^ *γ*D-crystallin solution at -2 °C, c) upon decompression (LLPS formation) and d) upon compression (vanishing of LLPS) at a constant *p*-jump amplitude of 1000 bar. The absorption data are normalized to their maximum values (1.0).


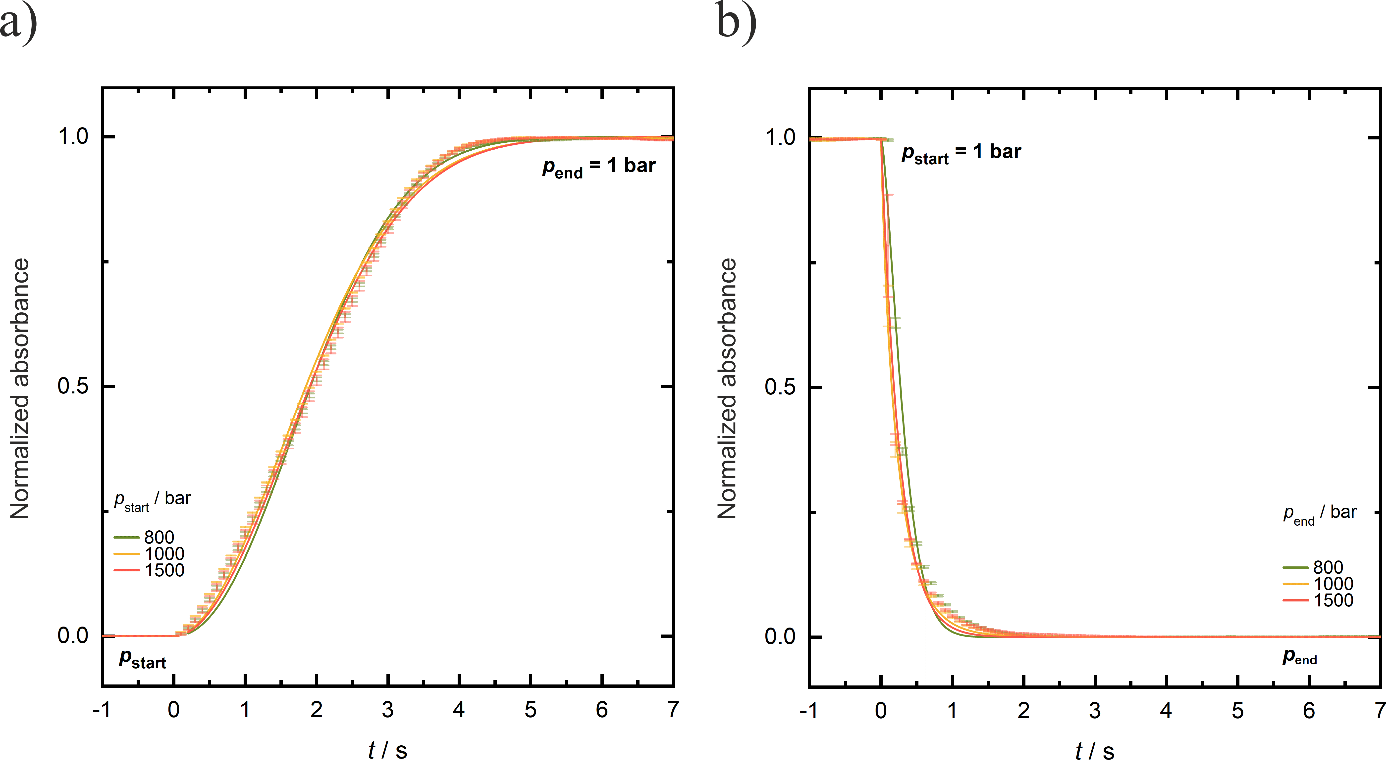


**Figure SI 6**. Effect of 10 wt% Ficoll on the kinetics of LLPS of a 50 mg mL^-1^ *γ*D-crystallin solution at 3 °C a) upon decompression (LLPS formation) and b) upon compression (vanishing of LLPS) at different *p*-jump amplitudes. The absorption data are normalized to their maximum values (absorbance = 1.0).

**Results of the Kinetic Analysis**

**Table SI 2.** Kinetic parameters obtained from the fits to the Johnson-Mehl-Avrami-Kolmogorov (JMAK) function to yield the Avrami exponent, *n*, and rate constant, *k*. The times *t*_tr_ and *t*_1/2_ were determined directly from the normalized absorption data and denote the overall transition time of the phase transformation and the half-time obtained from the point where the turbidity changed by 50%, respectively. The concentration of *γ*D-crystallin is 50 mg mL^-1^ and a temperature range from -2 to 11 °C was covered. Pressure-jump amplitudes of 500-2000 bar were used in both directions.

**SI 2.1**

| 0 M TMAO / -2.0 °C | | |  |  | |  |  |
| --- | --- | --- | --- | --- | --- | --- | --- |
| *P*_start_ / bar | *n* | | | *k* / s^-1^ | | *t*_tr_ / s | *t*_A ½_ / s |
| 500 | 1.99 ± 0.02 | | | 0.17 ± 0.00 | | 5.1 | 2.1 |
| 800 | 1.97 ± 0.02 | | | 0.17 ± 0.00 | | 5.3 | 2.1 |
| 1000 | 2.01 ± 0.03 | | | 0.16 ± 0.00 | | 5.5 | 2.1 |
| 1600 | 1.95 ± 0.03 | | | 0.18 ± 0.00 | | 5.3 | 2.1 |
|  |  | | |  | |  |  |
| 0 M TMAO / 0 °C |  | | |  | |  |  |
| *P*_start_ / bar | *n* | | | *k* / s^-1^ | | *t*_tr_ / s | *t*_A ½_ / s |
| 300 | 2.05 ± 0.06 | | | 0.17 ± 0.01 | | 5.4 | 2.0 |
| 500 | 2.03 ± 0.11 | | | 0.16 ± 0.02 | | 5.2 | 2.0 |
| 1000 | 2.05 ± 0.06 | | | 0.16 ± 0.01 | | 5.1 | 2.0 |
| 1500 | 2.15 ± 0.15 | | | 0.15 ± 0.03 | | 4.8 | 2.0 |
| 2000 | 2.05 ± 0.02 | | | 0.16 ± 0.00 | | 4.8 | 2.1 |
|  |  | | |  | |  |  |
| 0 M TMAO / 3.0 °C | |  | | |  |  |  |
| *P*_start_ / bar | | *n* | | | *k* / s^-1^ | *t*_tr_ / s | *t*_A ½_ /s |
| 300 | | 1.90 ± 0.03 | | | 0.24 ± 0.01 | 4.4 | 1.8 |
| 500 | | 1.88 ± 0.90 | | | 0.24 ± 0.02 | 4.7 | 1.8 |
| 1000 | | 1.87 ± 0.02 | | | 0.24 ± 0.00 | 4.1 | 1.8 |
| 1500 | | 1.69 ± 0.02 | | | 0.27 ± 0.01 | 4.5 | 1.8 |
| 2000 | | 1.87 ± 0.17 | | | 0.22 ± 0.04 | 4.1 | 1.8 |

**SI 2.2**

| 0 M TMAO / -2.0 °C | |  |  |  |  |
| --- | --- | --- | --- | --- | --- |
| *P*_end_ / bar | *n* | | *k* / s^-1^ | *t*_tr_ / s | *t*_A ½_ / s |
| 500 | 0.97 ± 0.64 | | 3.16 ± 2.27 | 1.5 | 0.2 |
| 800 | 0.86 ± 0.60 | | 3.21 ± 2.34 | 1.5 | 0.2 |
| 1000 | 0.71 ± 0.61 | | 3.54 ± 2.99 | 1.4 | 0.1 |
| 1600 | 0.68 ± 0.58 | | 3.42 ± 2.72 | 1.4 | 0.1 |
|  |  | |  |  |  |
| 0 M TMAO / 0 °C |  | |  |  |  |
| *P*_end_ / bar | *n* | | *k* / s^-1^ | *t*_tr_ / s | *t*_A ½_ / s |
| 300 | 1.85 ± 1.16 | | 3.11 ± 2.54 | 1.6 | 0.4 |
| 500 | 1.10 ± 0.07 | | 3.25 ± 0.23 | 1.4 | 0.2 |
| 1000 | 1.53 ± 0.10 | | 3.18 ± 0.25 | 1.6 | 0.3 |
| 1500 | 1.33 ± 0.84 | | 3.26 ± 2.58 | 1.4 | 0.3 |
|  |  | |  |  |  |
| 0 M TMAO / 3.0 °C | |  |  |  |  |
| *P*_end_ / bar | *n* | | *k* / s^-1^ | *t*_tr_ / s | *t*_A ½_ / s |
| 300 | 1.37 ± 0.48 | | 2.56 ± 0.96 | 1.7 | 0.3 |
| 500 | 0.87 ± 0.34 | | 2.81 ± 1.03 | 1.5 | 0.2 |
| 1000 | 1.00 ± 0.43 | | 3.56 ± 1.84 | 1.3 | 0.2 |
| 1500 | 0.66 ± 0.32 | | 3.08 ± 1.24 | 1.3 | 0.1 |

**SI 2.3**

| 0.3 M TMAO / 3.0 °C |  | |  |  |  |
| --- | --- | --- | --- | --- | --- |
| *P*_start_ / bar | *n* | | *k* / s^-1^ | *t*_tr_ / s | *t*_A ½_ / s |
| 600 | 1.75 ± 0.03 | | 0.17 ± 0.00 | 5.5 | 2.3 |
| 1000 | 1.75 ± 0.02 | | 0.18 ± 0.01 | 4.9 | 2.3 |
| 1500 | 1.73 ± 0.06 | | 0.19 ± 0.01 | 4.7 | 2.2 |
| 2000 | 1.79 ± 0.03 | | 0.18 ± 0.00 | 4.8 | 2.2 |
|  |  | |  |  |  |
| 0.3 M TMAO / 7.0 °C |  | |  |  |  |
| *P*_start_ / bar | *n* | | *k* / s^-1^ | *t*_tr_ / s | *t*_A ½_ / s |
| 600 | 1.81 ± 0.10 | | 0.19 ± 0.02 | 4.8 | 2.2 |
| 1000 | 1.73 ± 0.04 | | 0.19 ± 0.01 | 4.4 | 2.2 |
| 1500 | 1.90 ± 0.03 | | 0.16 ± 0.01 | 4.5 | 2.3 |
| 2000 | 1.76 ± 0.07 | | 0.18 ± 0.01 | 4.8 | 2.2 |
|  |  | |  |  |  |
| 0.3 M TMAO / 11.0 °C | |  |  |  |  |
| *P*_start_ / bar | *n* | | *k* / s^-1^ | *t*_tr_ / s | *t*_A ½_ / s |
| 600 | 1.83 ± 0.02 | | 0.21 ± 0.00 | 4.2 | 1.9 |
| 1000 | 1.84 ± 0.03 | | 0.19 ± 0.01 | 4.3 | 2.1 |
| 1500 | 1.93 ± 0.03 | | 0.18 ± 0.00 | 4.4 | 2.1 |
| 2000 | 1.76 ± 0.01 | | 0.19 ± 0.00 | 4.5 | 2.2 |

**SI 2.4**

| 0.3 M TMAO / 3.0 °C |  |  |  |  |
| --- | --- | --- | --- | --- |
| *P*_end_ / bar | *n* | *k* / s^-1^ | *t*_tr_ / s | *t*_A ½_ / s |
| 600 | 1.21 ± 0.74 | 3.53 ± 2.76 | 1.7 | 0.3 |
| 1000 | 0.83 ± 0.65 | 3.89 ± 3.53 | 1.5 | 0.1 |
| 1600 | 1.02 ± 0.70 | 3.95 ± 3.53 | 1.7 | 0.2 |
|  |  |  |  |  |
| 0.3 M TMAO / 7.0 °C |  |  |  |  |
| *P*_end_ / bar | *n* | *k* / s^-1^ | *t*_tr_ / s | *t*_A ½_ / s |
| 600 | 1.07 ± 0.70 | 3.31 ± 2.54 | 1.8 | 0.2 |
| 1000 | 0.56 ± 0.51 | 3.19 ± 2.20 | 1.6 | 0.1 |
| 1600 | 0.89 ± 0.59 | 3.03 ± 2.05 | 1.7 | 0.2 |
|  |  |  |  |  |
| 0.3 M TMAO / 11 °C |  |  |  |  |
| *P*_end_ / bar | *n* | *k* / s^-1^ | *t*_tr_ / s | *t*_A ½_ / s |
| 600 | 0.79 ± 0.48 | 2.56 ± 1.33 | 1.9 | 0.2 |
| 1000 | 0.68 ± 0.54 | 3.20 ± 2.27 | 1.5 | 0.1 |
| 1600 | 0.61 ± 0.48 | 2.97 ± 1.79 | 1.5 | 0.1 |

**SI 2.5**

| *p*_start_ = 1000 bar / 3.0 °C | |  |  |  |  |
| --- | --- | --- | --- | --- | --- |
| TMAO / M | *n* | | *k* / s^-1^ | *t*_tr_ / s | *t*_A ½_ / s |
| 0 | 1.87 ± 0.02 | | 0.24 ± 0.00 | 4.1 | 1.8 |
| 0.3 | 1.75 ± 0.02 | | 0.18 ± 0.00 | 4.9 | 2.3 |
| 0.5 | 2.01 ± 0.02 | | 0.15 ± 0.00 | 5.4 | 2.2 |

**SI 2.6**

| *P*_end_ = 1000 bar / 3.0 °C | |  |  |  |  |
| --- | --- | --- | --- | --- | --- |
| TMAO / M | *n* | | *k* / s^-1^ | *t*_tr_ / s | *t*_A ½_ / s |
| 0 | 1.00 ± 0.43 | | 3.56 ± 1.84 | 1.3 | 0.2 |
| 0.3 | 0.83 ± 0.65 | | 3.89 ± 3.53 | 1.5 | 0.1 |
| 0.5 | 0.83 ± 0.61 | | 3.37 ± 2.62 | 1.5 | 0.2 |

**SI 2.7**

| 0.2 M Urea / -2.0 °C |  |  |  |  |
| --- | --- | --- | --- | --- |
| *P*_start_ / bar | *n* | *k* / s^-1^ | *t*_tr_ / s | *t*_A ½_ / s |
| 500 | 1.98 ± 0.02 | 0.21 ± 0.00 | 5.1 | 1.9 |
| 800 | 1.92 ± 0.02 | 0.23 ± 0.00 | 5.5 | 1.8 |
| 1000 | 1.99 ± 0.02 | 0.20 ± 0.00 | 5.2 | 1.9 |
| 1600 | 1.92 ± 0.02 | 0.21 ± 0.00 | 5.2 | 1.9 |
|  |  |  |  |  |
| 0.2 M Urea / 2.0 °C |  |  |  |  |
| *P*_start_ / bar | *n* | *k* / s^-1^ | *t*_tr_ / s | *t*_A ½_ / s |
| 500 | 1.94 ± 0.02 | 0.26 ± 0.00 | 5.0 | 1.7 |
| 800 | 1.95 ± 0.02 | 0.26 ± 0.00 | 4.9 | 1.7 |
| 1000 | 1.95 ± 0.02 | 0.25 ± 0.00 | 4.8 | 1.7 |
| 1600 | 1.99 ± 0.02 | 0.23 ± 0.00 | 4.8 | 1.8 |

**SI 2.8**

| 0.2 M Urea / -2.0 °C |  |  |  |  |
| --- | --- | --- | --- | --- |
| *P*_end_ / bar | *n* | *k* / s^-1^ | *t*_tr_ / s | *t*_A ½_ / s |
| 500 | 0.92 ± 0.66 | 3.46 ± 2.82 | 1.5 | 0.2 |
| 800 | 0.83 ± 0.62 | 3.40 ± 2.70 | 1.5 | 0.2 |
| 1000 | 0.75 ± 0.56 | 3.14 ± 2.19 | 1.6 | 0.1 |
| 1600 | 0.72 ± 0.55 | 3.14 ± 2.18 | 1.5 | 0.1 |
|  |  |  |  |  |

| 0.2 M Urea / 2.0 °C |  |  |  |  |
| --- | --- | --- | --- | --- |
| *P*_end_ / bar | *n* | *k* / s^-1^ | *t*_tr_ / s | *t*_A ½_ / s |
| 500 | 0.90 ± 0.59 | 3.02 ± 2.02 | 1.7 | 0.2 |
| 800 | 0.83 ± 0.57 | 3.04 ± 2.04 | 1.6 | 0.2 |
| 1000 | 0.83 ± 0.57 | 3.03 ± 2.01 | 1.6 | 0.2 |
| 1600 | 0.92 ± 0.60 | 3.01 ± 2.01 | 1.7 | 0.2 |

**SI 2.9**

| *p*_start_ = 1000 bar / -2.0 °C | |  |  |  |  |
| --- | --- | --- | --- | --- | --- |
| Urea / M | *n* | | *k* / s^-1^ | *t*_tr_ / s | *t*_A ½_ / s |
| 0 | 2.01 ± 0.03 | | 0.16 ± 0.00 | 5.6 | 2.1 |
| 0.2 | 1.99 ± 0.02 | | 0.20 ± 0.00 | 5.3 | 1.9 |
| 0.3 | 2.07 ± 0.02 | | 0.17 ± 0.00 | 5.4 | 2.0 |

**SI 2.10**

| *P*_end_ = 1000 bar / -2.0 °C |  |  |  |  |
| --- | --- | --- | --- | --- |
| Urea / M | *n* | *k* / s^-1^ | *t*_tr_ / s | *t*_A ½_ / s |
| 0 | 0.71 ± 0.61 | 3.54 ± 2.99 | 1.4 | 0.1 |
| 0.2 | 0.75 ± 0.56 | 3.14 ± 2.19 | 1.6 | 0.1 |
| 0.3 | 0.73 ± 0.56 | 3.24 ± 2.36 | 1.7 | 0.1 |

**SI 2.11**

| 10 % wt Ficoll / 3.0 °C | |  |  |  |  |
| --- | --- | --- | --- | --- | --- |
| *P*_start_ / bar | *n* | | *k* / s^-1^ | *t*_tr_ / s | *t*_A ½_ / s |
| 800 | 2.14 ± 0.03 | | 0.17 ± 0.01 | 4.9 | 2.0 |
| 1000 | 1.92 ± 0.02 | | 0.21 ± 0.00 | 5.0 | 1.9 |
| 1500 | 1.97 ± 0.02 | | 0.20 ± 0.00 | 4.8 | 2.0 |
|  |  | |  |  |  |
| 10 % wt Ficoll / 7.0 °C | |  |  |  |  |
| *P*_start_ / bar | *n* | | *k* / s^-1^ | *t*_tr_ / s | *t*_A ½_ / s |
| 800 | 2.31 ± 0.04 | | 0.15 ± 0.01 | 4.8 | 2.0 |
| 1000 | 2.19 ± 0.03 | | 0.16 ± 0.01 | 4.8 | 2.0 |
| 1500 | 2.06 ± 0.03 | | 0.20 ± 0.01 | 5.0 | 1.9 |

**SI 2.12**

| 10 % wt Ficoll / 3.0 °C | |  |  |  |  |
| --- | --- | --- | --- | --- | --- |
| *P*_end_ / bar | *n* | | *k* / s^-1^ | *t* / s | *t*_A ½_ / s |
| 800 | 1.44 ± 0.57 | | 4.54 ± 1.76 | 1.8 | 0.3 |
| 1000 | 0.87 ± 0.26 | | 3.59 ± 1.07 | 1.5 | 0.2 |
| 1500 | 1.02 ± 0.61 | | 3.95 ± 1.86 | 1.5 | 0.2 |
|  |  | |  |  |  |
| 10 % wt Ficoll / 7.0 °C | |  |  |  |  |
| *P*_end_ / bar | *n* | | *k* / s^-1^ | *t* / s | *t*_A ½_ / s |
| 800 | 0.94 ± 0.64 | | 2.91 ± 1.92 | 1.5 | 0.2 |
| 1000 | 1.03 ± 0.14 | | 3.16 ± 1.57 | 1.6 | 0.2 |
| 1500 | 0.97 ± 0.32 | | 3.16 ± 1.62 | 1.6 | 0.2 |

**SI 2.13**

| 20 % wt Ficoll / 3.0 °C | |  |  |  |  |
| --- | --- | --- | --- | --- | --- |
| *P*_start_ / bar | *n* | | *k* / s^-1^ | *t*_tr_ / s | *t*_A ½_ / s |
| 800 | 1.84 ± 0.02 | | 0.21 ± 0.00 | 5.0 | 2.0 |
| 1000 | 2.10 ± 0.04 | | 0.17 ± 0.01 | 5.3 | 2.0 |
| 1500 | 2.03 ± 0.03 | | 0.19 ± 0.01 | 5.2 | 2.0 |
|  |  | |  |  |  |
| 20 % wt Ficoll / 7.0 °C | |  |  |  |  |
| *P*_start_ / bar | *n* | | *k* / s^-1^ | *t*_tr_ / s | *t*_A ½_ / s |
| 800 | 2.24 ± 0.03 | | 0.15 ± 0.01 | 4.9 | 2.0 |
| 1000 | 2.15 ± 0.03 | | 0.18 ± 0.01 | 5.0 | 2.0 |
| 1500 | 2.19 ± 0.03 | | 0.17 ± 0.01 | 4.7 | 2.0 |

**SI 2.14**

| 20 % wt Ficoll / 3.0 °C | |  |  |  |  |
| --- | --- | --- | --- | --- | --- |
| *P*_end_ / bar | *n* | | *k* / s^-1^ | *t* / s | *t*_A ½_ / s |
| 800 | 1.21 ± 0.27 | | 3.75 ± 1.23 | 1.7 | 0.3 |
| 1000 | 0.98 ± 0.18 | | 3.62 ± 1.83 | 1.5 | 0.2 |
| 1500 | 1.34 ± 0.48 | | 4.59 ± 2.91 | 1.5 | 0.2 |
|  |  | |  |  |  |
| 20 % wt Ficoll / 7.0 °C | |  |  |  |  |
| *P*_end_ / bar | *n* | | *k* / s^-1^ | *t* / s | *t*_A ½_ / s |
| 800 | 1.06 ± 0.54 | | 2.50 ± 1.10 | 2.2 | 0.3 |
| 1000 | 0.97 ± 0.40 | | 3.19 ± 1.59 | 1.7 | 0.2 |
| 1500 | 0.86 ± 0.43 | | 3.20 ± 1.60 | 1.6 | 0.2 |

**SI 2.15**

| *p*_start_ = 1000 bar / 3.0 °C | |  |  |  |  |
| --- | --- | --- | --- | --- | --- |
| Ficoll / % wt | *n* | | *k* / s^-1^ | *t*_tr_ / s | *t*_A ½_ / s |
| 0 | 1.93 ± 0.02 | | 0.24 ± 0.00 | 4.1 | 1.8 |
| 10 | 1.92 ± 0.02 | | 0.21 ± 0.00 | 5.0 | 1.9 |
| 20 | 2.10 ± 0.04 | | 0.17 ± 0.01 | 5.3 | 2.0 |

**SI 2.16**

| *P*_end_ = 1000 bar / 3.0 °C |  |  |  |  |
| --- | --- | --- | --- | --- |
| Ficoll / % wt | *n* | *k* / s^-1^ | *t*_tr_ / s | *t*_A ½_ / s |
| 0 | 0.71 ± 0.61 | 3.54 ± 2.99 | 3.0 | 0.1 |
| 10 | 0.87 ± 0.26 | 3.59 ± 1.07 | 1.5 | 0.2 |
| 20 | 0.98 ± 0.18 | 3.62 ± 1.83 | 1.5 | 0.2 |
